# Comparative pain relief and functional outcomes following robotic-assisted versus conventional total knee arthroplasty: a GRADE-assessed meta-analysis with meta-regression of randomized controlled trials

**DOI:** 10.1007/s11701-026-03934-0

**Published:** 2026-09-25

**Authors:** Omar Abdelaziz, Ziad G. Zayed, Ahmed Mohamed Altukhy, Salem Waleed Salem, Mohamed A. Hanafy, Rofida Hamdy, Haytham Abdelrahman, Abdalla M. Hadhoud

**Affiliations:** 1 Faculty of Medicine, Alexandria University, Alexandria, 21526 Egypt; 2 Faculty of Medicine, Benha University, Benha, 13511 Egypt; 3 College of Human Medicine, Benha University, Benha, 13511 Egypt; 4 Faculty of Medicine, Tanta University, Tanta, 31527 Egypt; 5 Cairo University Hospitals, Cairo, 12662 Egypt

**Keywords:** Robotic-assisted total knee arthroplasty, Conventional total knee arthroplasty, Postoperative pain, Functional recovery, Patient satisfaction, Blood loss, Meta-analysis

## Abstract

Although robotic-assisted total knee arthroplasty (RA-TKA) is increasingly adopted for its potential to improve surgical precision, its impact on functional outcomes compared with conventional TKA (C-TKA) remains unclear. Existing syntheses are limited by pooling observational and randomized data, focusing on absolute postoperative scores without evaluating recovery trajectories, and lacking rigorous exploration of heterogeneity. To overcome these limitations, we conducted a methodologically rigorous meta-analysis of randomized controlled trials (RCTs) only, incorporating comprehensive meta-regression, platform-specific subgroup analyses, and assessment of evidence certainty using the Grading of Recommendations Assessment, Development, and Evaluation (GRADE) framework. PubMed, Web of Science, Scopus, the Cochrane Library, and Google Scholar (*pages 1–50*) were comprehensively searched from their inception up to December 27, 2025. Data were pooled using random-effects meta-analysis and Hartung-Knapp adjustments. Meta-regression of different moderators and subgroup analyses by robotic platforms were performed, when feasible, to identify sources of heterogeneity. A total of 24 RCTs encompassing 3,425 patients were included. The pooled analyses demonstrated no significant differences between RA-TKA and C-TKA for change in Western Ontario and McMaster Universities Osteoarthritis Index (WOMAC) function score (MD 0.14; 95% CI − 5.88 to 6.16; *p* = 0.96), change in functional Knee Society Score (KSS) (MD 0.51; 95% CI − 3.43 to 4.44; *p* = 0.78), or change in Oxford Knee Score (OKS) (MD 2.07; 95% CI − 6.82 to 10.96; *p* = 0.51). Although a subgroup analysis revealed a significant improvement in OKS at 6 months only (MD 2.25; *p* = 0.01), this effect did not reach the minimal clinically important difference (MCID) and was not sustained at other time points. No significant differences were observed for range of motion, change in VAS pain score, KSS satisfaction, or length of hospital stay. Operative time was significantly longer for RA-TKA (MD 22.34 min; 95% CI 13.66 to 31.02; *p* < 0.01), though this estimate should be interpreted with caution given the very high heterogeneity (I² = 98.53%) and the likelihood that it includes cases performed during the early learning curve of robotic adoption. Trial Sequential Analysis suggested that this finding is unlikely to be attributable to random error, though the required information size was not reached. RA-TKA and C-TKA provide comparable functional improvement, pain relief, and most perioperative outcomes. The most consistent signal is a prolonged operative time with RA-TKA favoring conventional surgery, though the precise magnitude remains uncertain and may be influenced by the learning curve. The certainty of evidence is low to very low across all assessed outcomes, highlighting the need for cautious interpretation. Current evidence does not support the routine adoption of RA-TKA over C-TKA for TKA. Future adequately powered, multicenter RCTs with long-term follow-up and consistent reporting of surgeon experience are required to definitively clarify the role of RA-TKA in contemporary practice.

**Supplementary Information:**

The online version contains supplementary material available at https://doi.org/10.1007/s11701-026-03934-0.

## Introduction

Over one million total knee arthroplasties (TKA) are executed annually across the United States, with current projections estimating this volume to surpass three million cases by the year 2040 [1]. The literature supports its effectiveness; however, conventional TKA (C-TKA) still faces challenges with optimal component alignment and soft-tissue balancing, as indicated by the 20% of patients who report dissatisfaction after the procedure [2]. These technical challenges have prompted the development of robotic-assisted TKA (RA-TKA) that combines 3D imaging, computer navigation, and robotic assistance to facilitate alignment and potentially improve patient satisfaction [3, 4]. The underlying hypothesis of RA-TKA is that more accurate technical alignment of the components will result in superior patient-centered outcomes [5]. However, the clinical significance of this enhanced surgical precision remains controversial, with studies showing inconsistent results between radiographic improvements and functional outcomes [6, 7]. This disconnect illustrates the importance of focusing on patient-centered outcomes, rather than radiographic outcomes alone.

Consequently, post-surgical functional recovery serves as a primary benchmark for determining procedural success. This recovery is routinely quantified using validated, standardized evaluation tools, most notably KSS, OKS, and WOMAC. These measures assess patients’ ability to execute fundamental activities of daily life, general function, and joint range of motion, all of which reflect physical independence and quality of life [8]. The nature and extent of functional recovery after TKA are key variables in the rehabilitation process, the level of postoperative care provided, and the patient’s long-term well-being. Postoperative pain, using a visual analog scale (VAS), constitutes another key metric and can significantly impact recovery and patient satisfaction [9]. Pain that is inadequately managed or uncontrolled after surgery may delay functional rehabilitation, delay the return to usual activities, and be a source of patient dissatisfaction and revision surgery [7, 9]. The relationship between surgical technique and pain reporting has been demonstrated but remains incompletely understood. However, enhanced accuracy may theoretically optimize joint biomechanics and reduce aberrant stress distribution. Patient satisfaction is the paramount patient-centered endpoint that encompasses several domains, including pain relief, improvement in function, realization of pre-operative expectations, and overall perception of surgical success. Given that one-fifth of TKA patients are dissatisfied despite arguably objective radiographic success, determining strategies to maximize this critical endpoint during surgery is a research priority [7, 10].

Despite the growing number of meta-analyses which compare the two techniques in arthroplasty practices, several critical gaps remain [11–13]. First, many prior syntheses have pooled randomized and observational studies, potentially introducing confounding and obscuring true treatment effects derived from high-level evidence. However, this study is restricted to randomized controlled trials (RCTs), thereby ensuring more robust causal inference and minimizing the confounding inherent in observational data. Second, while previous meta-analyses have largely focused on absolute postoperative outcomes, such measures do not adequately capture the magnitude of patient improvement. In contrast, our study uniquely concentrates on change‑from‑baseline scores, which more accurately reflect the degree of recovery and account for potential differences in baseline patient characteristics across studies. This distinction is clinically meaningful, as two patients with identical postoperative scores may have experienced vastly different magnitudes of improvement depending on their preoperative status. Third, we carried out comprehensive meta-regression analyses to discover the cause of heterogeneity across multiple clinically relevant covariates, an approach that has been limited or absent in prior work. This allows for a more rigorous exploration of potential effect modifiers and sources of between-study variability. Fourth, we conducted subgroup analyses by specific robotic platform, when feasible, allowing identification of potential system-specific effects rather than treating robotic-assisted TKA as a homogeneous intervention. Fifth, we performed Trial Sequential Analysis (TSA) for operative time, an outcome characterized by very high heterogeneity, to assess whether the observed difference is robust against random error, thereby providing a more definitive evaluation of this important outcome. Finally, we applied the GRADE framework to provide a transparent and clinically interpretable evaluation of the strength of the findings. Collectively, these methodological innovations provide a more rigorous and clinically meaningful synthesis of the current evidence base and address key limitations of previous meta-analyses.

## Methods

### Search strategy

This review strictly adhered to the PRISMA 2020 recommendations [14] and was registered on PROSPERO (*CRD420261301318*). Two authors independently carried out a thorough search of five major biomedical databases, covering all eligible publications available through December 27, 2025: PubMed, Web of Science, Scopus, the Cochrane Library, and Google Scholar (*pages 1–50*). In addition, we supplemented our search by hand-searching the reference lists of included studies and relevant reviews to search for any additional eligible records not captured by the electronic database searches. The detailed search strategy is available in supplementary material as **Supplementary Table 1**.

## Inclusion and exclusion criteria

This review was limited to RCTs to avoid the confounding present in observational data and to give a rigorous causal assessment of the treatment impact. The population included adult patients scheduled to undergo total knee arthroplasty for any indication. The intervention was robotic-assisted TKA, with any available robotic system. Comparators were conventional TKA, performed using manual or conventional techniques without robotic assistance. To warrant inclusion, studies were required to have data on one or more of these clinical endpoints: **Pain and Function Measures**: Postoperative pain severity via change in VAS; standardized functional metrics, including changes in OKS, KSS, and WOMAC function subscales. **Surgical and Perioperative Outcomes**: Joint mobility evaluated through range of motion (ROM); length of stay (LOS); operative time (minutes); estimated intraoperative or cumulative blood loss (mL); post-surgical wound drainage volume (mL); perioperative hemoglobin fluctuations; systemic inflammatory indicators; and validated patient satisfaction scores. Exclusion criteria were non-RCTs, case series, study protocols, or abstracts; studies not comparing RA-TKA to C-TKA or lacking a control group; studies not reporting relevant clinical outcomes. Eligibility assessment was carried out independently by two investigators using Rayyan [15].

## Data extraction

Independently, data were extracted by two investigators by a pre‑piloted Microsoft Excel spreadsheet. To comprehensively capture functional recovery, we extracted change‑from‑baseline scores for key functional outcomes (e.g., WOMAC, KSS, OKS), as these measures reflect the magnitude of improvement over time and account for potential differences in baseline patient characteristics across studies. Unlike absolute postoperative scores, which merely indicate the final functional level achieved, change‑from‑baseline scores are more sensitive to differences in the rate and magnitude of early recovery, providing a more meaningful assessment of whether a technique accelerates improvement and/or alters the ultimate functional outcome.

To address potential unit-of-analysis issues, outcomes reported per knee were carefully evaluated. One included RCT (*Song et al.*, 28) involved simultaneous bilateral procedures and reported data at the knee level. Patient-level data were extracted when available; otherwise, knee-level data were included under an assumption of independence, which may underestimate variance and overestimate precision. To evaluate this approach, leave-one-out sensitivity analyses were undertaken for all outcomes addressed in this study. Additionally, to systematically assess and prevent double‑counting of participants from the same RCT cohort across multiple publications (e.g.,* studies with different follow‑up durations or shared authorship*), we constructed supplementary tables (**Supplementary Tables S2–S6**) for studies with potential patient overlap. Each table presents key information for related publications, including study ID, total sample size with breakdown by intervention group (RA‑TKA/C‑TKA), setting or hospital, patient recruitment period, brief inclusion criteria, and key outcomes addressed, to evaluate the extent of potential overlap. Where substantial overlap was identified, we applied a hierarchical approach: for each unique patient cohort, we retained only the publication with the most comprehensive or most extended follow‑up for a given outcome in the primary meta‑analysis. In cases where multiple publications from the same trial reported distinct, non‑overlapping outcomes, data were extracted from each relevant publication, carefully ensuring no patient overlap in the pooled analysis. This strategy ensured that no studies with potential patient overlap were pooled together in the same analysis, thereby maintaining the independence of observations and the integrity of the pooled estimates.

## Quality assessment

The internal validity and methodological quality of the selected trials were appraised independently by two reviewers using the Cochrane Risk of Bias 2 (RoB-2) framework [16]. Evaluations targeted five primary domains: the randomization process, deviations from assigned protocols, missing outcome data, objective measurement of endpoints, and selective reporting behavior. Each domain was subsequently classified as presenting low risk, some concerns, or a high risk of bias, with any coder discrepancies settled by a third author via consensus. The certainty of evidence for selected critical outcomes was evaluated using the Grading of Recommendations Assessment, Development and Evaluation (GRADE) guidelines [17].

### Statistical analysis

Meta-analyses, using random‑effects models with the Restricted Maximum‑Likelihood (REML) estimator, were carried out using R (version 4.5.2), and 95% CIs were calculated using the Hartung‑Knapp adjustment. Effect sizes for continuous outcomes were pooled as mean differences (MDs). Heterogeneity was evaluated using Cochrane’s Q and I² statistics. When heterogeneity was detected, leave‑one‑out sensitivity analyses were undertaken to check the extent to which other results would be affected. In addition, we performed leave‑one‑out sensitivity analyses for all outcomes with more than two studies, including those with high heterogeneity, to evaluate the robustness of the pooled estimates and identify potential outlier studies. Moreover, publication bias was assessed using funnel plots, and for outcomes with 10 or more studies, Egger’s regression test was performed. Influence analyses were evaluated using Baujat plots to identify studies that contributed to heterogeneity and significantly influenced the pooled effect sizes. All statistical tests were two‑tailed, and p‑values < 0.05 were considered statistically significant. To explore the impact of different robotic platforms, we performed subgroup analyses for outcomes for which data were available from at least two studies for each robotic system. This was done to address potential heterogeneity and provide a more granular understanding of the results. To understand sources of heterogeneity, pre‑specified random‑effects meta‑regressions were conducted on three key outcomes: change in WOMAC function score, change in KSS function, and operative time. We chose these outcomes for their clinical importance, observed heterogeneity, and enough contributing studies. Four covariates were examined for each outcome: mean age (years), mean BMI (kg/m²), proportion of female participants (%), and follow‑up duration (months). For change‑from‑baseline outcomes, when the standard deviation (SD) of the change was not directly reported, we used the Meta‑Analysis Accelerator tool [18]. By providing the means and SDs before and after the intervention, along with the correlation coefficient, the tool calculates the mean change and standard deviation of the change.

A Trial Sequential Analysis (TSA) was undertaken to control for random errors and evaluate the robustness of the evidence for operative time, using TSA software (Copenhagen Trial Unit, Denmark). A two‑sided type I error (α) of 5% and a statistical power (1‑β) of 80% were applied [19–21]. The O’Brien‑Fleming alpha‑spending function was used to construct the trial sequential monitoring boundaries. The required information size (RIS) was estimated using the mean difference (MD) from the meta‑analysis and the model-variance-based method to correct for heterogeneity. To derive a more conservative and reliable variance estimate, we excluded studies judged to have a high risk of bias and calculated the pooled variance directly from the remaining trials, thereby reducing the influence of potentially flawed studies on the RIS estimate. The cumulative Z‑curve was plotted against the RIS, and the evidence was considered conclusive if the curve crossed the sequential monitoring boundaries before it reached the RIS.

## Results

### Search and screening

**Fig. 1:**
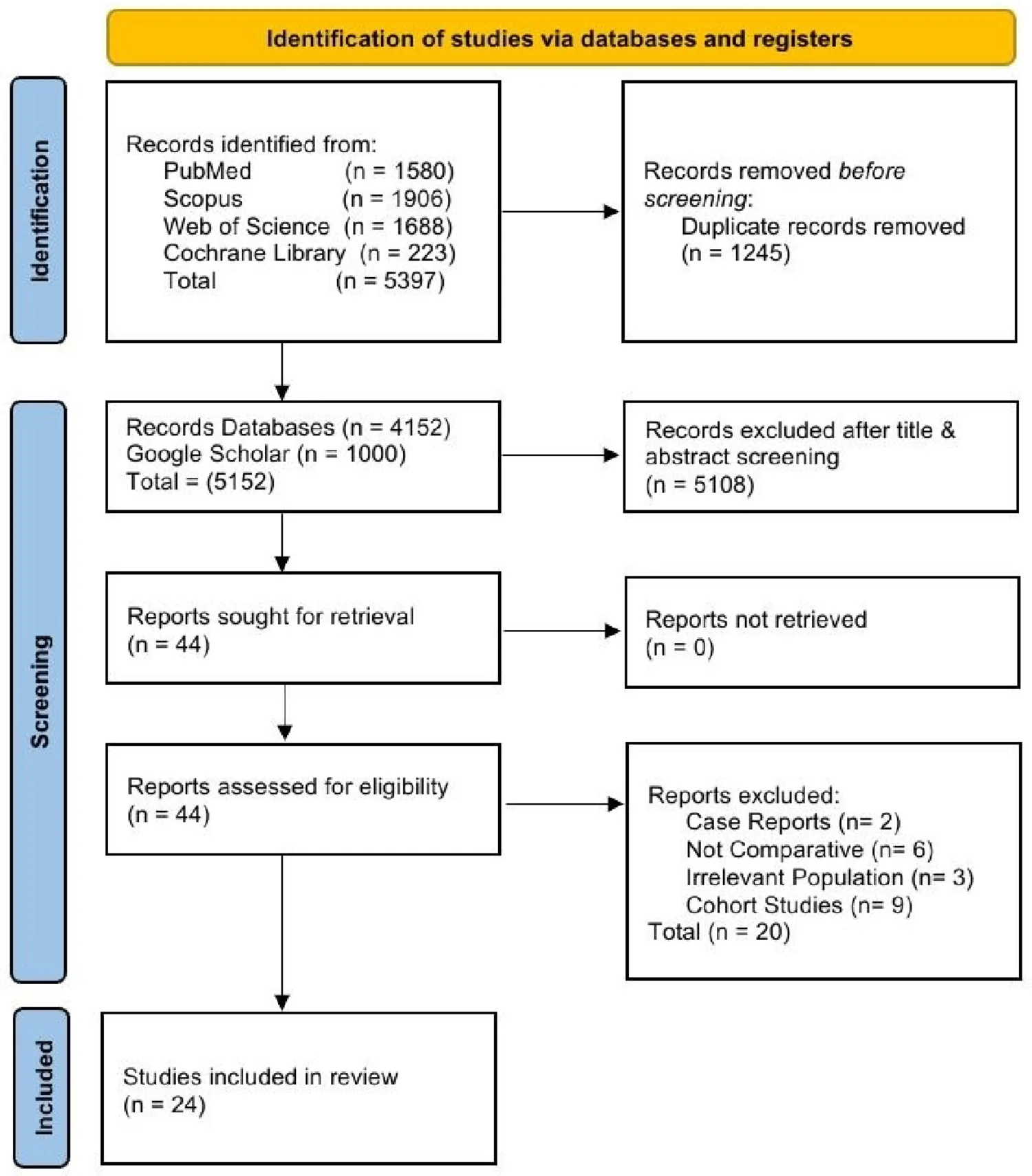
PRISMA flowchart of the selection process.

The literature search, completed on December 27, 2025, identified a total of 5,397 records across the four electronic databases (PubMed: 1,580; Scopus: 1,906; Web of Science: 1,688; Cochrane Library: 223). Following de‑duplication, 4,152 records remained for initial screening. In addition, 1000 records were identified from Google Scholar (*pages 1–50*) for title and abstract screening. After title and abstract screening, 44 articles were selected for full‑text screening. Following full‑text assessment, 20 articles were excluded for the following reasons: case reports (*n* = 2), not comparative studies (*n* = 6), irrelevant population (*n* = 3), non‑randomized study design (*n* = 9). The remaining 24 studies met the inclusion criteria and were included in the quantitative analysis. (Fig. 1).

## Summary of studies included

Twenty-four RCTs met the inclusion criteria, comprising an aggregate cohort of 3,425 patients. The included research demonstrated a broad and diverse geographical distribution. A significant concentration of studies originated from Asia, specifically China, South Korea, Thailand, and Singapore. European centers also made substantial contributions, with studies from the United Kingdom, Belgium, Russia, Poland, and Norway. This broad geographical scope enhances the generalizability of the review’s conclusions on different healthcare systems and patient populations. The publication dates ranged from 2011 to 2025, capturing over a decade of technological and clinical evolution in the field. There was considerable heterogeneity in the sample sizes, ranging from small pilot cohorts of 20 patients to landmark, large-scale trials enrolling over 1300 patients. The primary cause for surgery was osteoarthritis (OA) of the knee joint. A detailed summary of each study’s population and key characteristics is meticulously presented in Table 1.

**Table 1:** Summary and baseline characteristics of included studies

Study	Country	Total Sample Size	Mean Age (years) (RA-TKA / C-TKA)	BMI (kg/m²) (RA-TKA / C-TKA)	Male Percentage (%) (RA-TKA / C-TKA)
Adamska et al., 2022 [41]	Poland	215	67.45 ± 7.30 / 65.0 ± 8.2	25.66 ± 3.11 / 26.0 ± 3.17	38.1 / 44.1
Airapetov et al., 2023 [42]	Russia	20	60.47 ± 20.64 / 61.8 ± 24.08	NR	40.0 / 30.0
Bollars et al., 2025 [43]	Belgium	180	64.5 ± 8.9 / 65.8 ± 8.6	29.7 ± 4.9 / 29.4 ± 5.3	46.7 / 45.6
Clement et al., 2023 [35]	UK	100	66.8 ± 8.7 / 66.7 ± 9.6	31.7 ± 5.5 / 31.3 ± 6.8	52.0 / 42.0
Clement et al., 2025 [36]	UK	40	64.5 ± 7.3 / 63.8 ± 6.3	31 ± 5.8 / 32.2 ± 3.4	45.45 / 38.89
Fontalis et al., 2022 [37]	UK	30	67.9 ± 8.6 / 68.7 ± 9.6	27.0 ± 3.0 / 27.5 ± 3.7	40.0 / 46.7
Geng et al., 2024 [38]	China	130	68.1 ± 5.0 / 67.4 ± 5.5	NR	24.6 / 32.3
Geng et al., 2025 [39]	China	139	67.5 ± 7.2 / 68.1 ± 6.9	27.8 ± 4.1 / 28.2 ± 4.5	82.2 / 77.3
Kim et al., 2020 [6]	South Korea	1348	60 ± 7 / 61 ± 8	28 ± 9 / 29 ± 8	17.6 / 18.8
Li et al., 2021 [40]	China	150	68.0 ± 7.97 / 69.0 ± 6.00	26.9 ± 3.26 / 27.4 ± 3.38	17.8 / 19.5
Liow et al., 2014 [22]	Singapore	60	67.5 ± 8.6 / 68.3 ± 7.7	27.5 ± 3.8 / 27.2 ± 4.9	NR
Liow et al., 2016 [23]	Singapore	60	67.5 ± 8.6 / 68.3 ± 7.7	27.5 ± 3.8 / 27.2 ± 4.9	NR
Liu et al., 2024 [24]	China	88	72.0 ± 5.38 / 72.0 ± 5.35	25.63 ± 3.69 / 26.1 ± 3.75	26.8 / 25.5
Lychagin et al., 2024 [25]	Russia	118	67.7 ± 10.2 / 66.5 ± 8.7	31.4 ± 2.6 / 32.1 ± 4.1	21.4 / 24.2
Ren et al., 2025 [26]	China	66	66.8 ± 5.8 / 66.8 ± 5.1	27.4 ± 4.0 / 26.3 ± 3.0	18.8 / 35.3
Skåden et al., 2025 [27]	Norway	51	65.7 ± 3.7 / 67.7 ± 3.9	NR	53.8 / 40.0
Song et al., 2011 [28]	South Korea	30	67.0 ± 6.3 / 67.0 ± 6.3	27.0 ± 6.5 / 27.0 ± 6.5	0 / 0
Song et al., 2013 [29]	South Korea	100	66.1 ± 7.1 / 64.8 ± 5.3	26.3 ± 2.7 / 26.2 ± 3.9	8.0 / 10.0
Thiengwittayaporn et al., 2021 [30]	Thailand	152	69.0 ± 8.3 / 69.1 ± 7.3	28.0 ± 4.9 / 27.7 ± 4.6	8.0 / 19.5
Tian et al., 2022 [31]	China	123	68.17 ± 7.59 / 68.84 ± 7.12	25.62 ± 3.08 / 26.64 ± 3.33	21.0 / 24.6
Vermue et al., 2025 [32]	Belgium	60	65.0 ± 12.0 / 64.0 ± 8.0	30.0 ± 5.0 / 30.0 ± 4.0	27 / 27
Xu et al., 2022-1 [33]	China	33	66.6 ± 3.7 / 67.3 ± 3.5	25.6 ± 3.3 / 25.5 ± 2.9	17.6 / 18.8
Xu et al., 2022-2 [34]	China	72	64.5 ± 5.3 / 63.4 ± 7.2	26.2 ± 3.8 / 26.4 ± 3.1	29.7 / 20.0
Yuan et al., 2024 [44]	China	60	65.2 ± 6.4 / 65.4 ± 8.0	27.4 ± 3.0 / 28.4 ± 3.6	32.1 / 12.5

Abbreviations: C-TKA: Conventional TKA;RA-TKA: Robotic-Assisted TKA;NR: Not Reported

The interventional characteristics revealed a diverse landscape of modern TKA practice. A broad spectrum of robotic systems was utilized for RA-TKA, including haptic-guided systems (e.g., MAKO), imageless handheld systems (e.g., NAVIO), and active autonomous platforms (e.g., ROBODOC). In contrast, C-TKA procedures consistently used manual instrumentation with intramedullary femoral and extramedullary tibial guides. Further heterogeneity was noted in the choice of prosthesis (e.g., posterior-stabilized vs. cruciate-retaining), anesthesia protocols (general vs. regional), and tourniquet application strategies (continuous use, selective use, or no use at all). This variability in surgical technique and perioperative management provides essential context for interpreting the findings.

### Risk of bias assessment

**Fig. 2:**
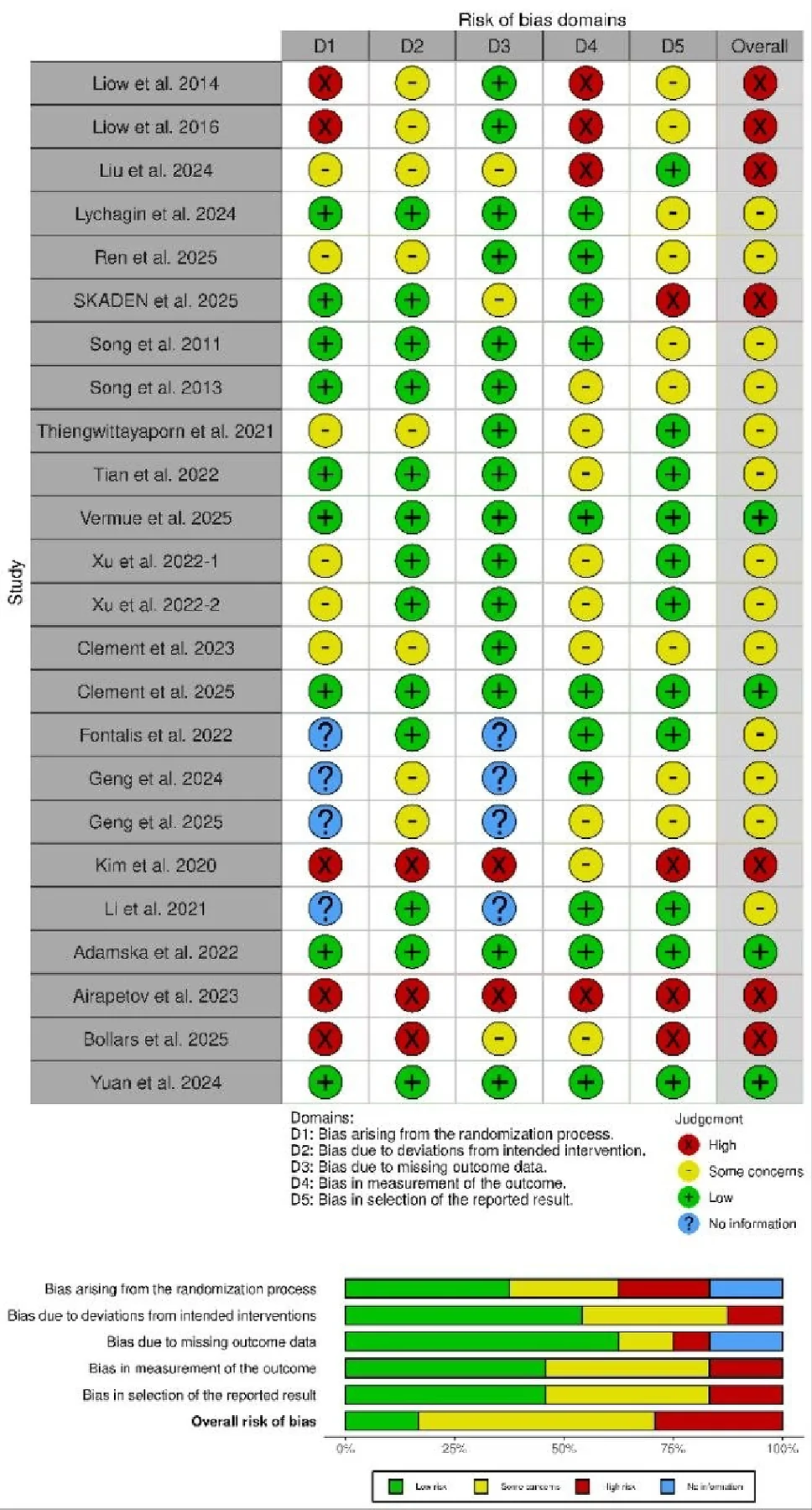
Assessment of the included studies' risk of bias (ROB)

To evaluate methodological rigor, we used the Cochrane Risk of Bias-2 (RoB-2) tool. A total of twenty-four studies were assessed, revealing variable methodological quality. Among the 24 studies, four were judged to have a low overall risk of bias, thirteen showed some concerns, and seven were found to have a high overall risk of bias. These trials, rated as low risk, demonstrated clearly reported randomization methods, proper allocation concealment, adequate blinding, and complete data reporting, thereby providing high internal validity. In contrast, seven studies were rated as having a high overall risk of bias. Familiar sources of bias among these trials included inadequate randomization, lack of blinding, selective outcome reporting, and incomplete data handling. The remaining thirteen studies were judged to have some concerns. These concerns primarily resulted from incomplete reporting of randomization procedures, unclear blinding, or partial outcome data, which introduced a moderate degree of uncertainty. (Fig. 2)

### Assessment of evidence certainty

The certainty of the evidence for the evaluated outcomes, as assessed using the GRADE framework, is summarized in Table 2. The evidence for change in WOMAC at 3‑months and 1‑year, as well as change in OKS at 1‑year, was rated as very low certainty, primarily downgraded due to serious risk of bias and very serious or extremely serious imprecision, with wide confidence intervals crossing the null effect. Similarly, the evidence for change in KSS at 1‑year and change in VAS pain at 1‑year was judged as very low certainty, downgraded for serious risk of bias, serious to very serious inconsistency, and serious to extremely serious imprecision. The evidence for change in KSS at 3‑months was rated as low certainty, limited by serious risk of bias and serious imprecision, though this outcome was considered clinically important. Operative time, also rated as very low certainty, showed a statistically significant increase with robotic‑assisted TKA, but was downgraded due to serious risk of bias, very serious inconsistency, and serious imprecision; nonetheless, it was deemed an important outcome. Intraoperative blood loss was rated with very low certainty, downgraded for serious risk of bias and extremely serious imprecision, with confidence intervals that included both clinically meaningful harm and benefit. Overall, all outcomes were supported by low to very-low certainty evidence, indicating that the true effect estimates remain uncertain and should be interpreted with caution.

**Table 2:** Summary of Findings (SoF) table – GRADE assessment of certainty of evidence

Certainty assessment							№ of patients		Effect		Certainty	Importance
№ of studies	Study design	Risk of bias	Inconsistency	Indirectness	Imprecision	Other considerations	RA-TKA	C-TKA	Relative (95% CI)	Absolute (95% CI)		
**Change in WOMAC at 3 months follow-up**												
5	randomised trials	serious	not serious	not serious	very serious	none	232	236	-	MD **1.47 lower** (5.85 lower to 2.91 higher)	⨁◯◯◯ Very low	NOT IMPORTANT
**Change in WOMAC at 1-year follow-up**												
3	randomised trials	serious	serious	not serious	extremely serious	none	73	71	-	MD **0.22 higher** (31.93 lower to 32.37 higher)	⨁◯◯◯ Very low	NOT IMPORTANT
**Change in KSS at 3 months follow-up**												
8	randomised trials	serious	not serious	not serious	serious	none	432	438	-	MD **1.24 higher** (2.89 lower to 5.36 higher)	⨁⨁◯◯ Low	IMPORTANT
**Change in KSS at 1-year follow-up**												
2	randomised trials	serious	very serious	not serious	extremely serious	none	118	121	-	MD **6.76 higher** (87.33 lower to 100.84 higher)	⨁◯◯◯ Very low	NOT IMPORTANT
**Change in OKS at 1-year follow-up**												
2	randomised trials	serious	not serious	not serious	very serious	none	105	98	-	MD **7.05 higher** (3.67 lower to 17.77 higher)	⨁◯◯◯ Very low	NOT IMPORTANT
**Change in VAS pain at 1-year follow-up**												
3	randomised trials	serious	very serious	not serious	serious	none	265	189	-	MD **0.45 lower** (3.09 lower to 2.18 higher)	⨁◯◯◯ Very low	NOT IMPORTANT
**Operative time (minutes)**												
14	randomised trials	serious	very serious	not serious	serious	none	1426	1347	-	MD **22.34 higher** (13.66 higher to 31.02 higher)	⨁◯◯◯ Very low	IMPORTANT
**Intraoperative blood loss (mL)**												
7	randomised trials	serious	not serious	not serious	extremely serious	none	936	939	-	MD **4.89 higher** (11.46 lower to 21.25 higher)	⨁◯◯◯ Very low	NOT IMPORTANT

***Abbreviations: CI***: *confidence interval;****MD***: *mean difference*

### Primary outcomes

**Fig. 3:**
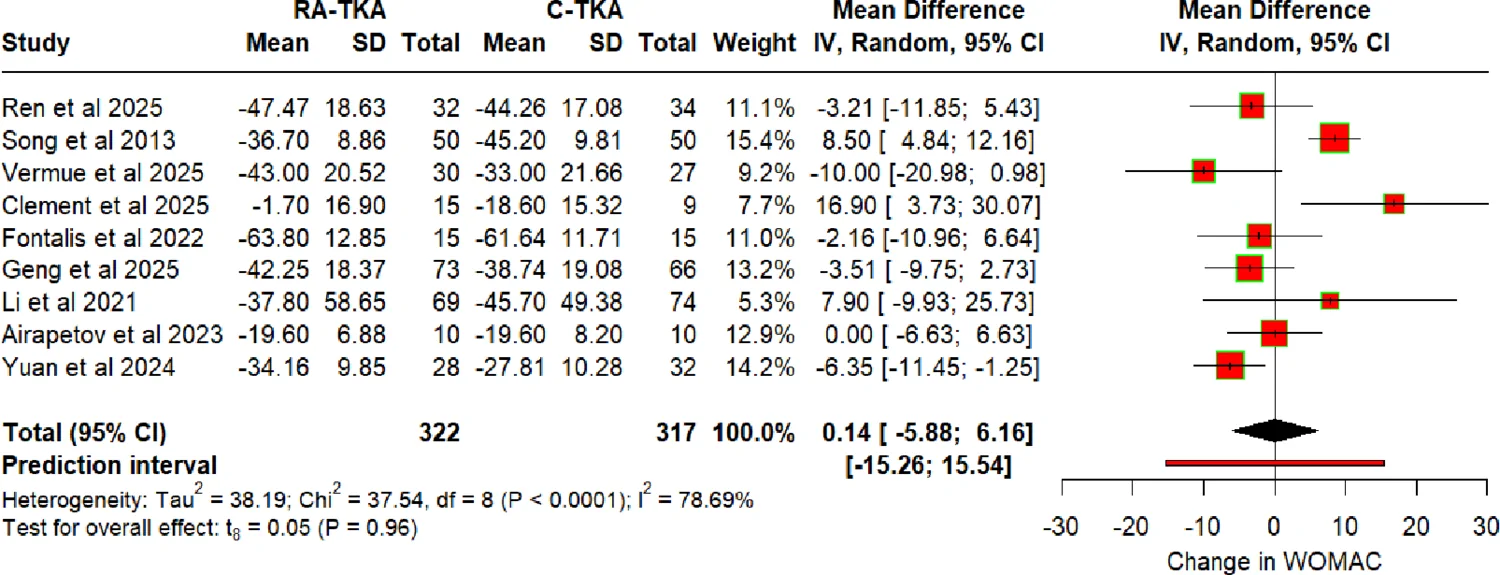
Forest plot of postoperative WOMAC function score changes between RA-TKA and C-TKA at final follow-up

**Fig. 4:**
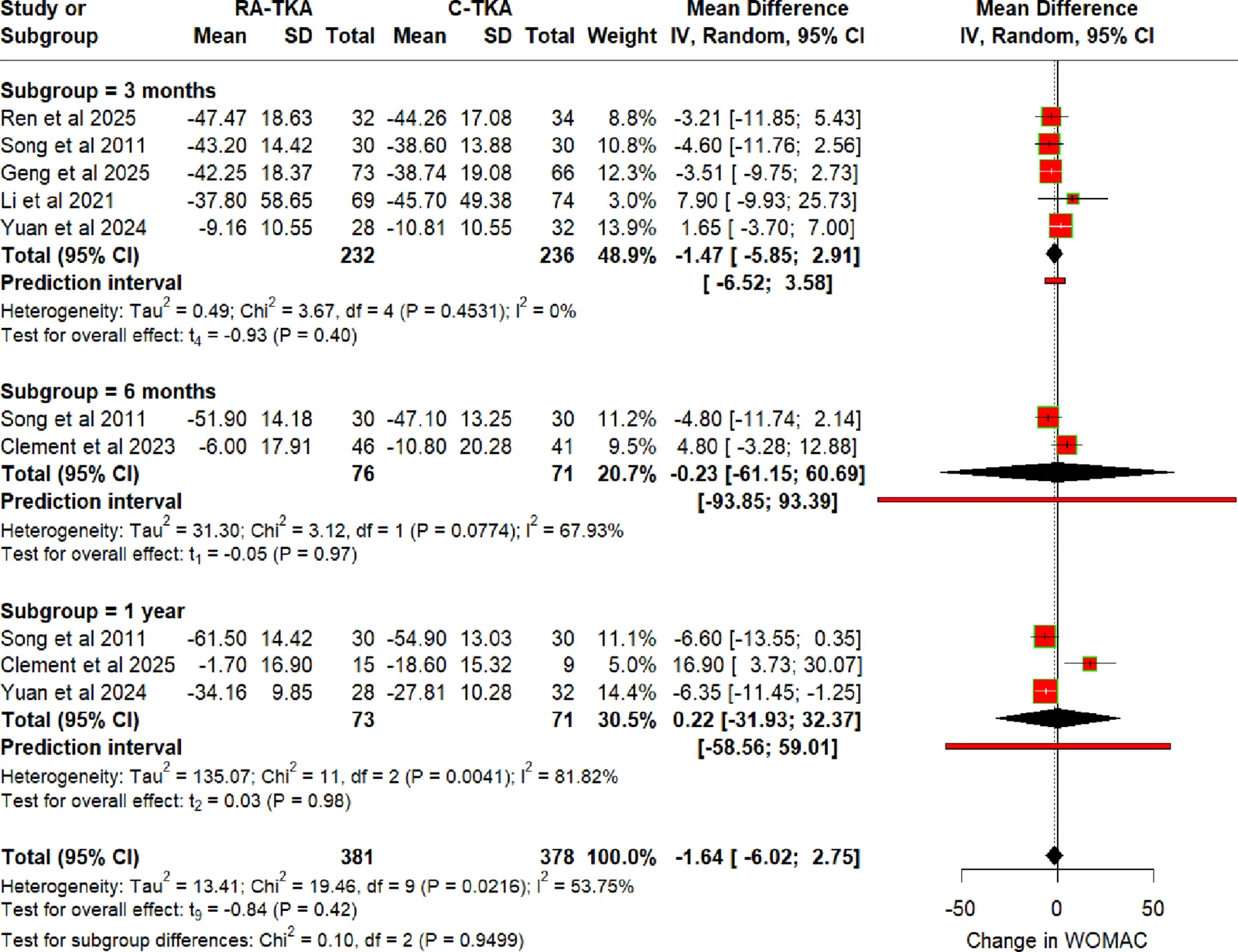
Forest plot of subgroup analysis of changes in WOMAC function score between RA-TKA and C-TKA across distinct follow-up intervals

The pooled analysis of the change in WOMAC-function score (*scale 0–100*, 9 studies, *n* = 639; RA-TKA = 322, C-TKA = 317) demonstrated no significant difference between the two groups (MD 0.14; 95% CI − 5.88 to 6.16; *p* = 0.96; I² = 78.69%). (Fig. 3) A subgroup analysis by follow-up revealed that the MD demonstrated no significant difference at 3-month, 6-month, and 1-year follow-up durations. (Fig. 4) Furthermore, leave-one-out sensitivity analysis showed that the overall result remained non-significant for all scenarios.

**Fig. 5:**
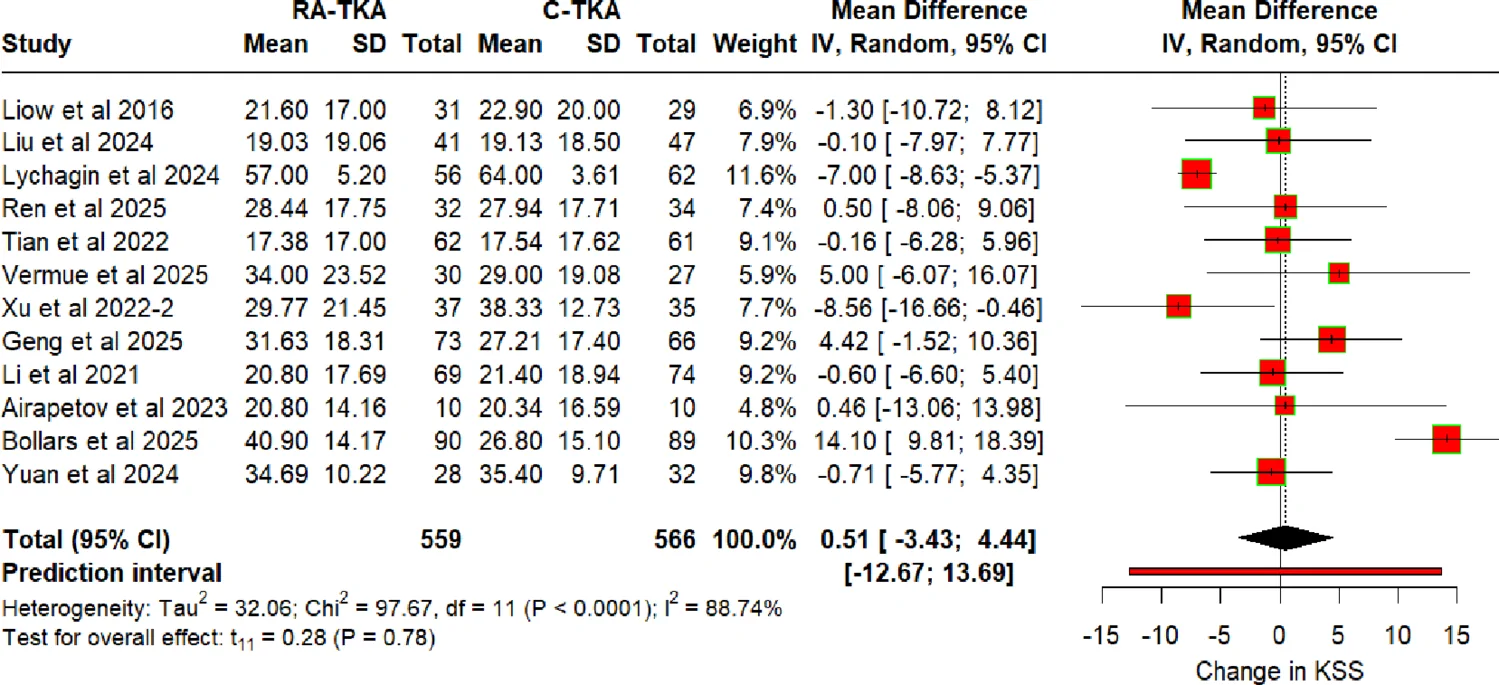
Forest plot of postoperative KSS function score changes between RA-TKA and C-TKA at final follow-up

**Fig. 6:**
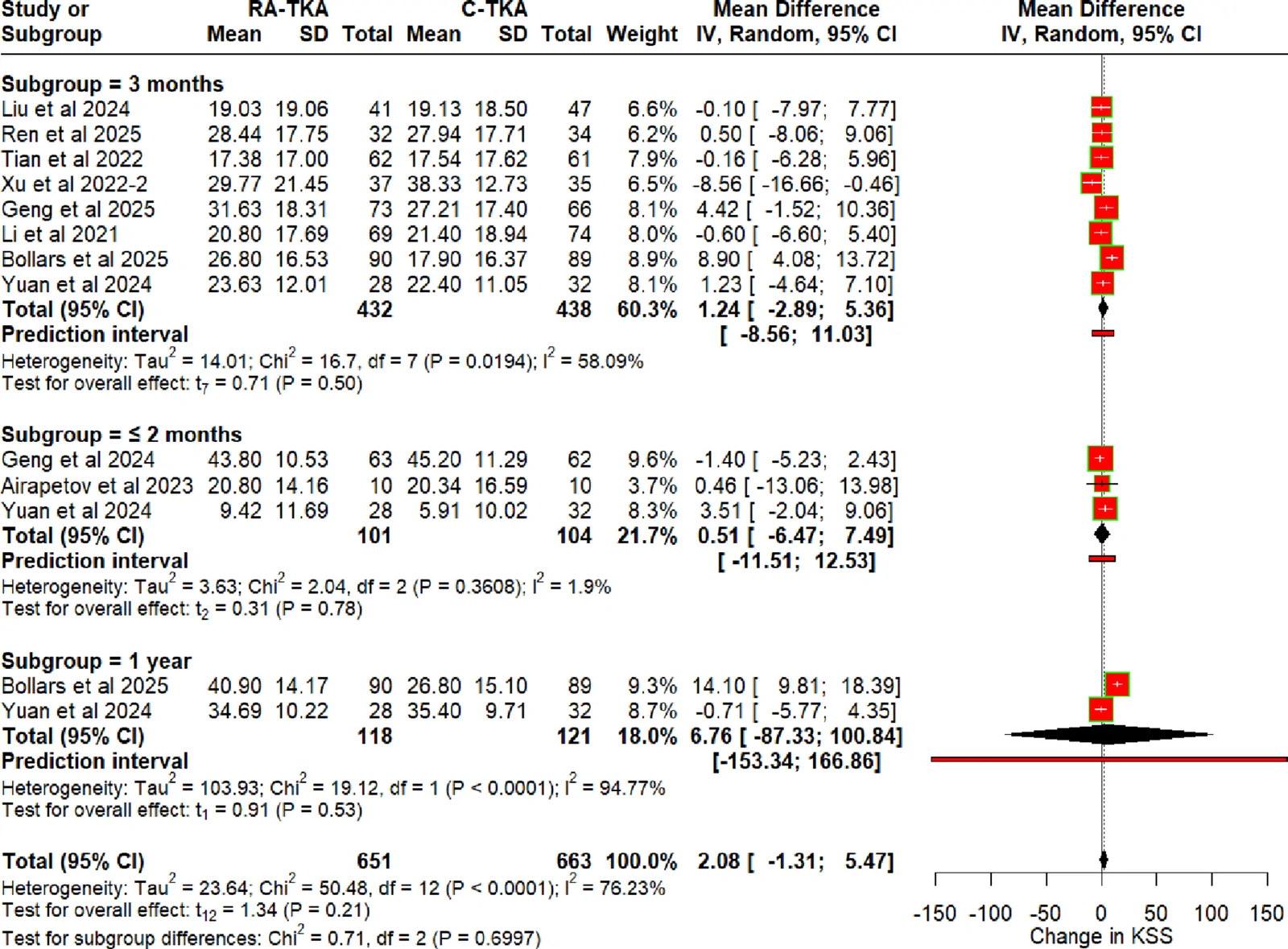
Forest plot of subgroup analysis of changes in KSS function score between RA-TKA and C-TKA across distinct follow-up intervals

**Fig. 7:**
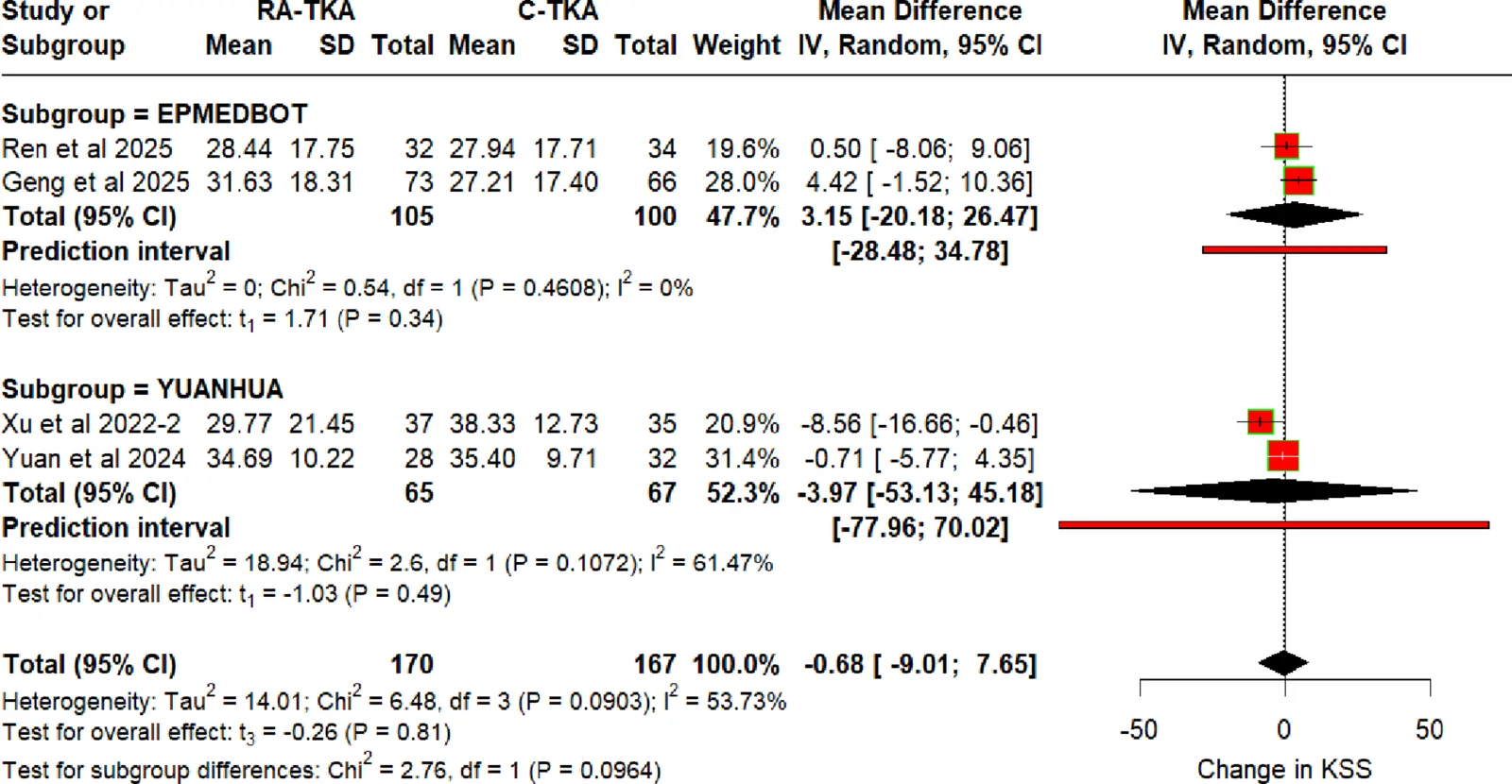
Forest plot showing the subgroup analysis by robotic platform of the change in KSS function score between RA-TKA and C-TKA at the last follow-up

The pooled analysis of the change in KSS function score (*scale 0–100*, 12 studies, *n* = 1,125; RA-TKA = 559, C-TKA = 566) did not reveal a significant difference between the two surgical approaches (MD 0.51; 95% CI − 3.43 to 4.44; *p* = 0.78; I² = 88.74%). (Fig. 5) This finding remained non-significant in all leave-one-out sensitivity analyses, supporting the null result. Furthermore, all follow-up points demonstrated no significant differences. (Fig. 6) Another subgroup analysis by robotic platforms revealed no significant difference with EPMEDBOT (MD 3.15; *p* = 0.34) or YUANHUA (MD -3.97; *p* = 0.49). (Fig. 7)

**Fig. 8:**
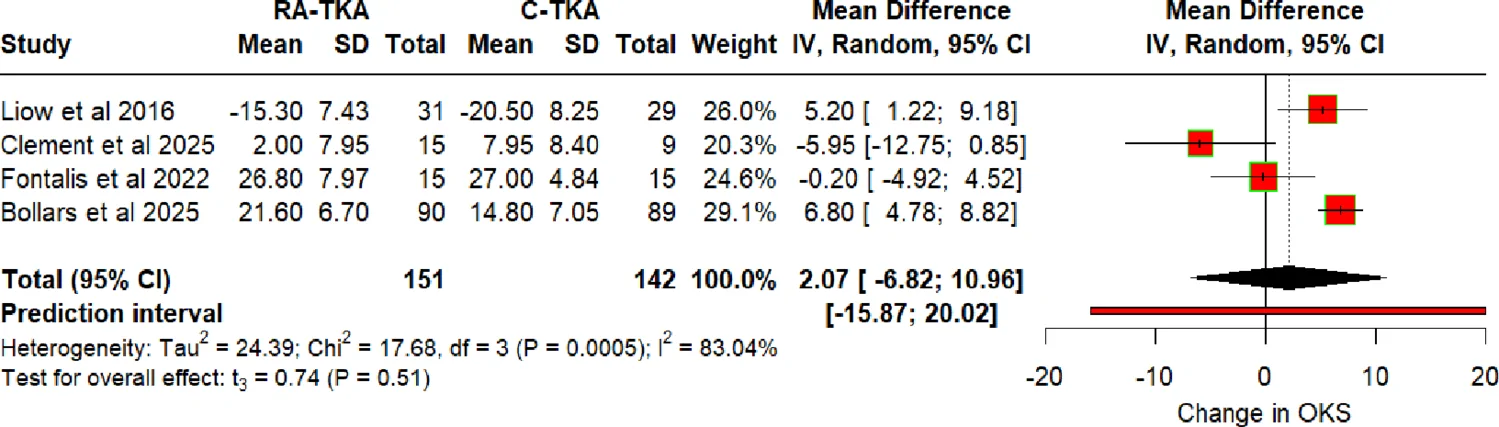
Forest plot of postoperative OKS changes between RA-TKA and C-TKA at final follow-up

**Fig. 9:**
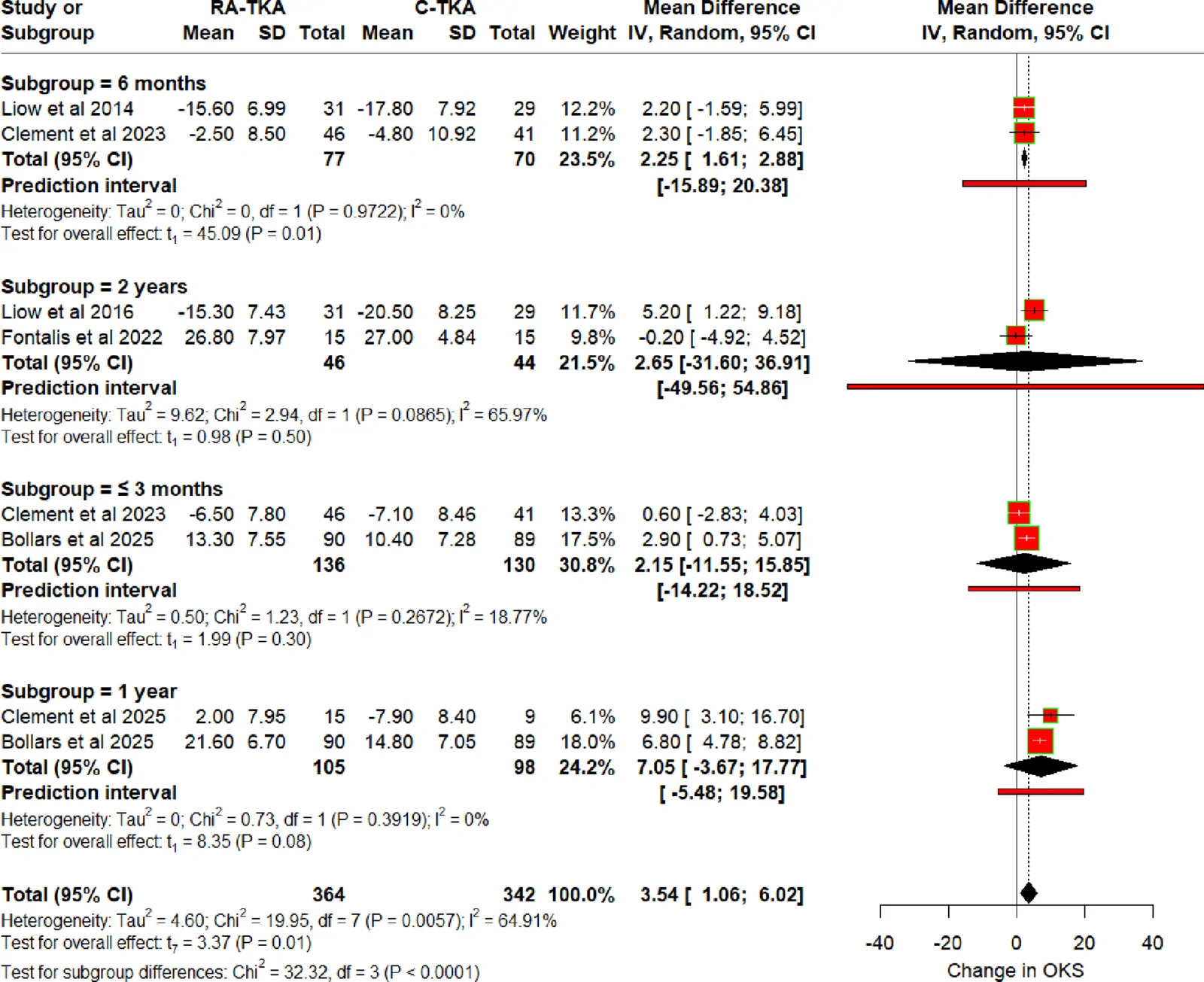
Forest plot of subgroup analysis of changes in OKS score between RA-TKA and C-TKA across distinct follow-up intervals

The meta-analysis of change in OKS (4 studies, *n* = 293; RA-TKA = 151, C-TKA = 142) demonstrated no statistically significant improvement (MD 2.07; 95% CI -6.82 to 10.96; *p* = 0.51; I² = 83.04%). (Fig. 8) This finding remained non-significant in all leave-one-out sensitivity analyses. A subgroup analysis by follow-up intervals revealed a significant difference at 6 months only (MD 2.25; 95% CI 1.61 to 2.88; *p* = 0.01; I² = 0.00%). However, this observed effect does not meet the minimal clinically important difference (MCID). In addition, no significant change was observed at any other follow-ups, supporting the fragility of this temporary effect. (Fig. 9)

**Fig. 10:**
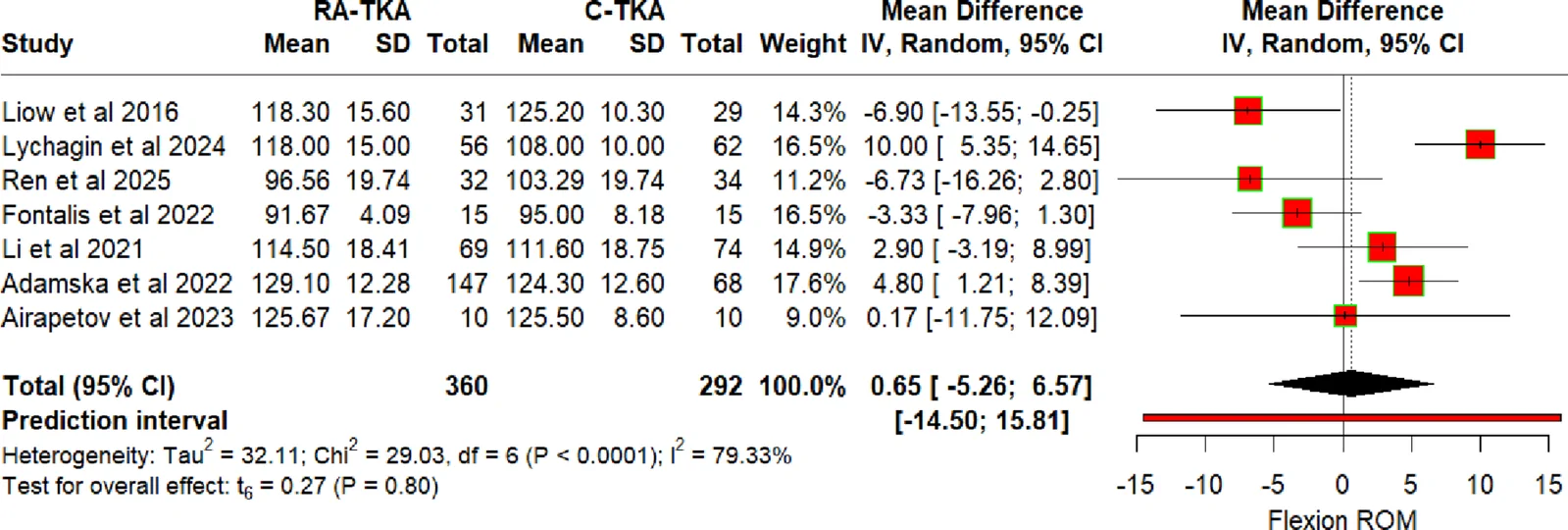
Forest plot of postoperative flexion ROM between RA-TKA and C-TKA at final follow-up

**Fig. 11:**
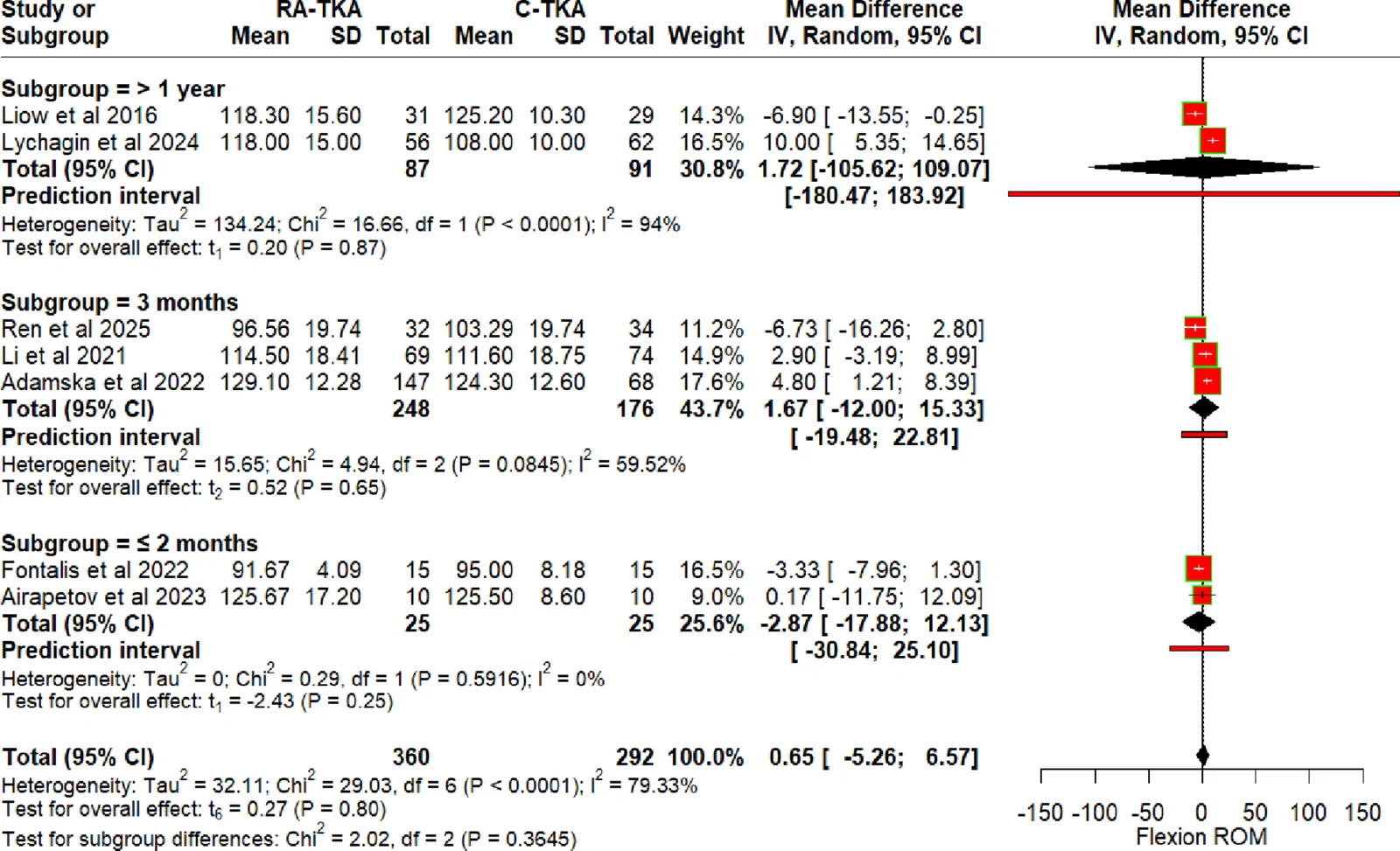
Forest plot of subgroup analysis of flexion ROM between RA-TKA and C-TKA across distinct follow-up intervals

**Fig. 12:**
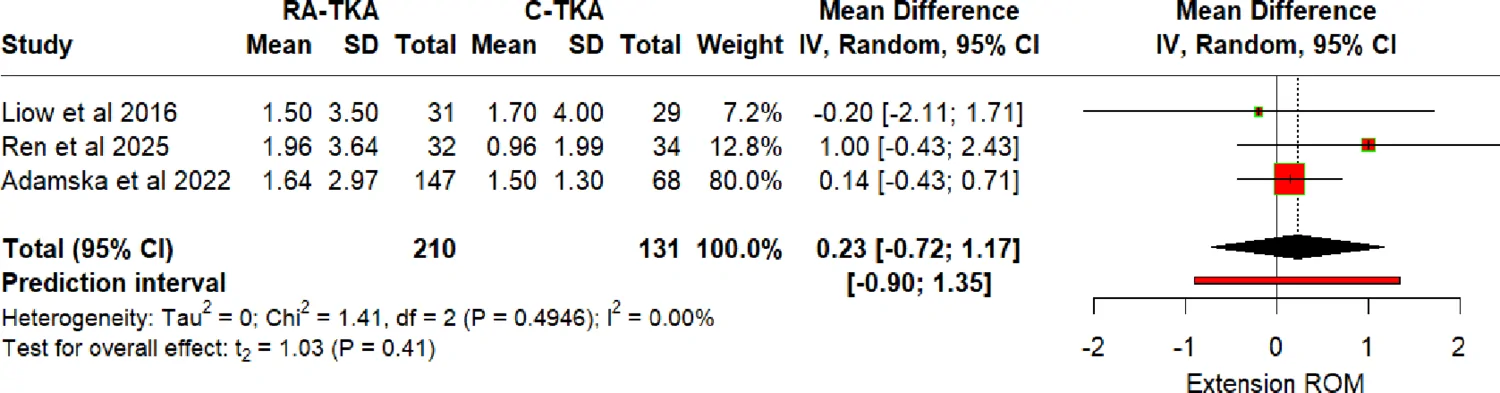
Forest plot of postoperative extension ROM between RA-TKA and C-TKA at final follow-up

The pooled analysis showed no differences in postoperative ROM between RA-TKA and C-TKA. The pooled mean difference for flexion (7 studies, *n* = 652; RA-TKA = 360, C-TKA = 292) was 0.65° (95% CI − 5.26 to 6.57; *p* = 0.80; I² = 79.33%). (Fig. 10) This finding remained non-significant in all leave-one-out sensitivity analyses. Furthermore, all follow-up points demonstrated no significant differences, supporting the null effect. (Fig. 11) For extension ROM, the pooled analysis showed no differences (3 studies, *n* = 341; RA-TKA = 210, C-TKA = 131), with a mean difference of 0.23° (95% CI − 0.72 to 1.17; *p* = 0.41; I² = 0.00%). (Fig. 12) Using leave-one-out sensitivity analysis, the pooled MD remained not significant in all cases after omitting each study.

**Fig. 13:**
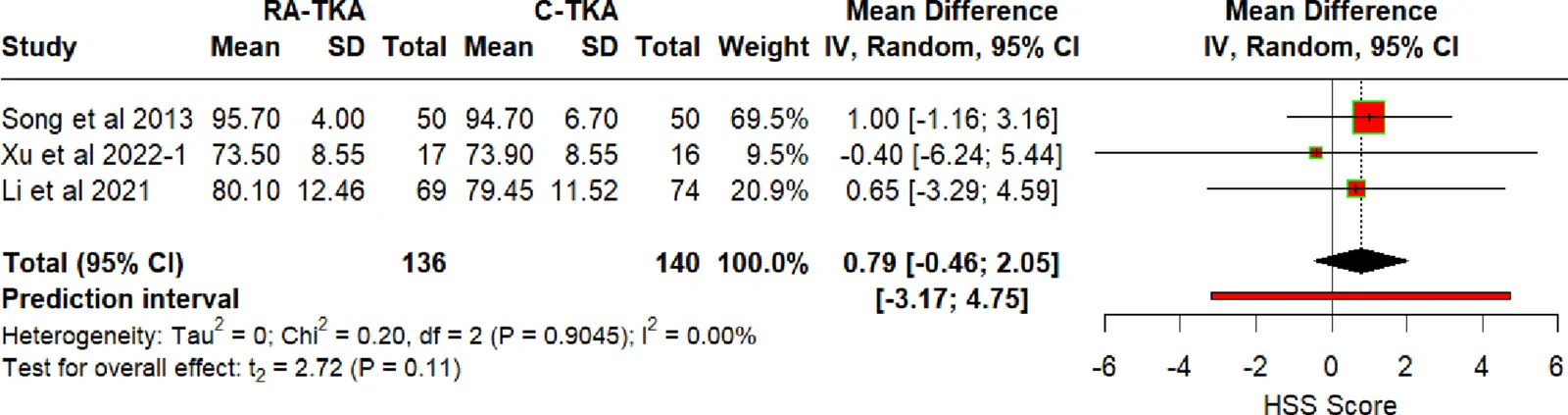
Forest plot of postoperative HSS score between RA-TKA and C-TKA at final follow-up

The analysis of postoperative Hospital for Special Surgery (HSS) score (3 studies, *n* = 276; RA-TKA = 136, C-TKA = 140) demonstrated no significant difference (MD 0.79; 95% CI -0.46 to 2.05; *p* = 0.11; I² = 0.00%). (Fig. 13) In a leave-one-out sensitivity analysis, the difference remained not significant in all cases after omitting each study.

**Fig. 14:**
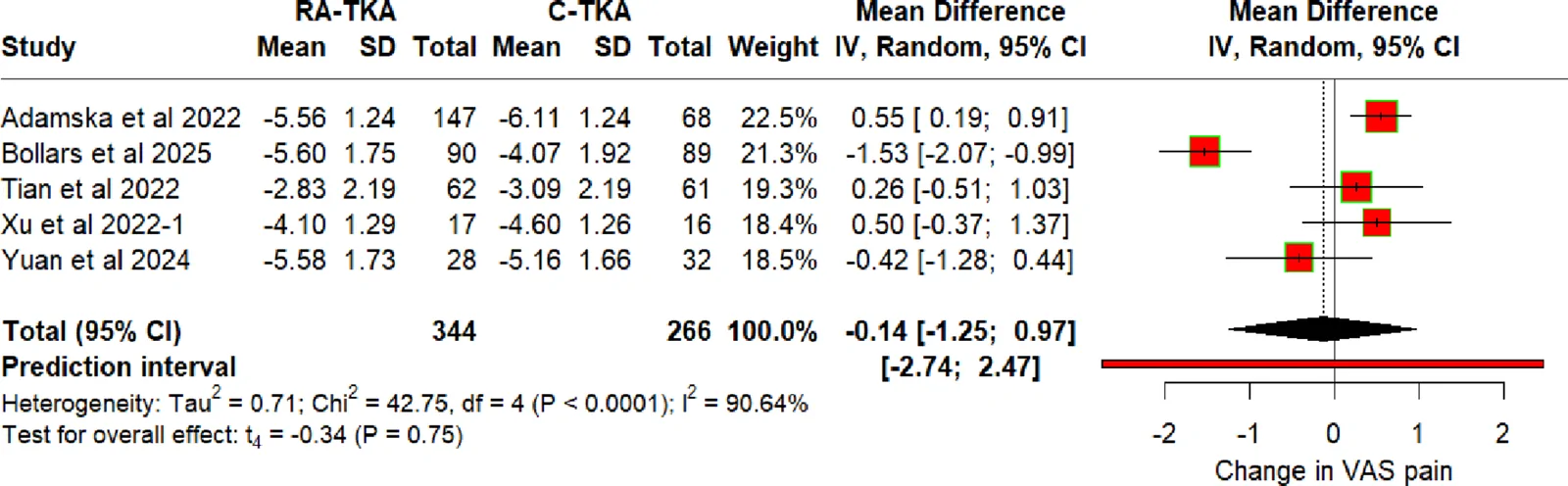
Forest plot of postoperative VAS pain score changes between RA-TKA and C-TKA at final follow-up

**Fig. 15:**
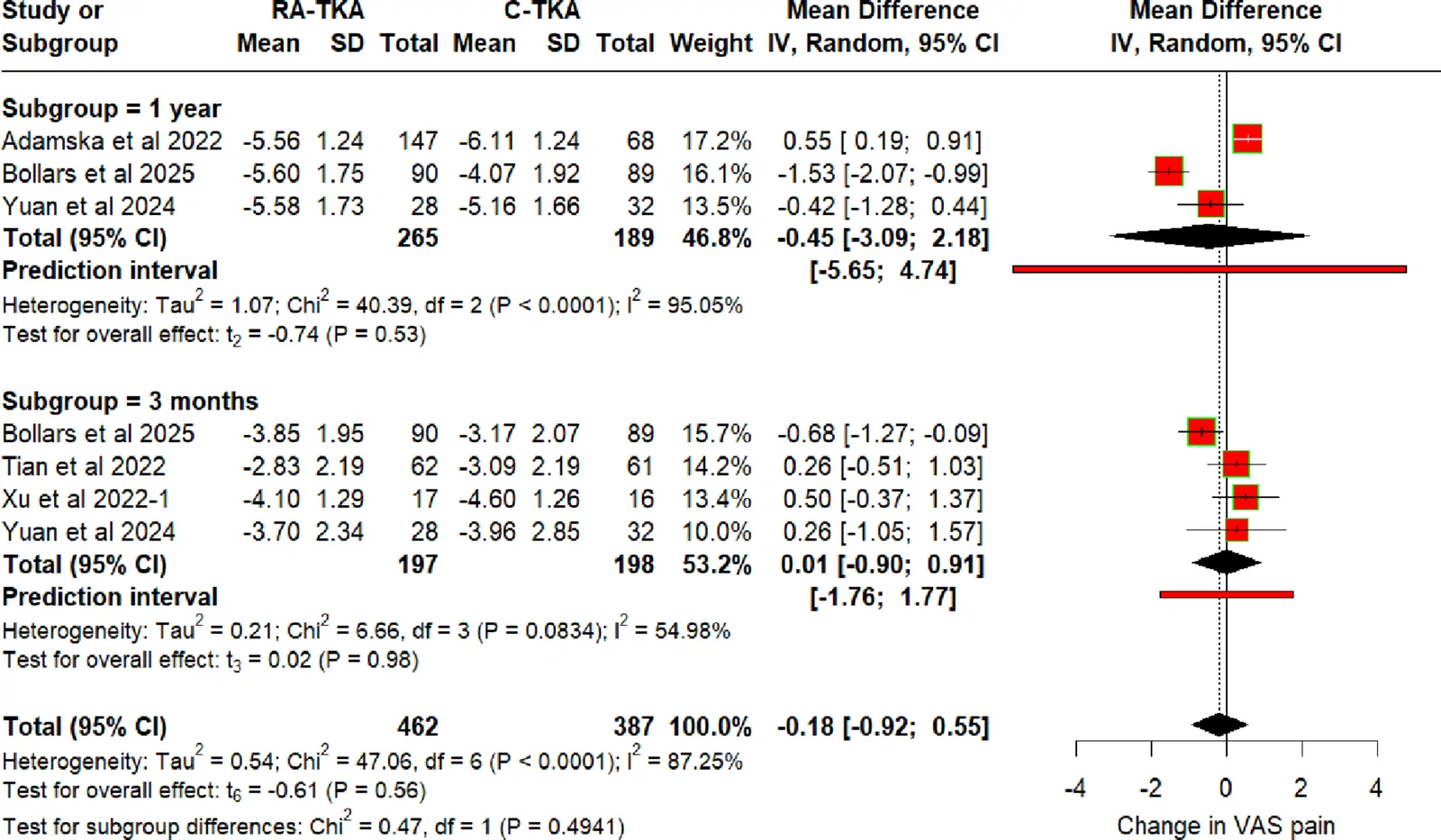
Forest plot of subgroup analysis of changes in VAS pain score between RA-TKA and C-TKA across distinct follow-up intervals

**Fig. 16:**
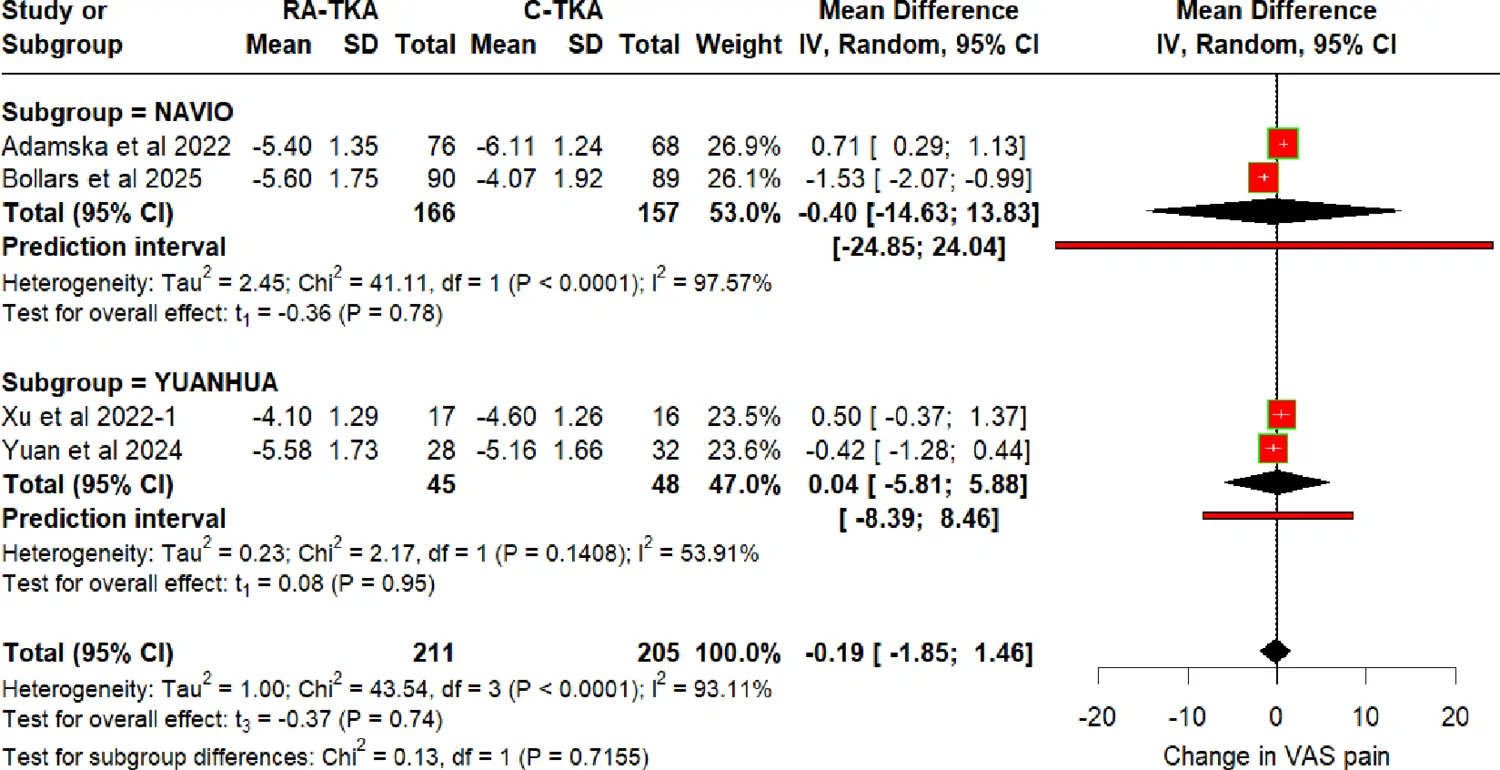
Forest plot showing the subgroup analysis by robotic platform of the change in VAS pain between RA-TKA and C-TKA at the last follow-up

The meta-analysis of change in VAS pain score (5 studies, *n* = 610; RA-TKA = 344, C-TKA = 266), *measured on a 0–10 scale*, demonstrated no differences between the 2 techniques (MD -0.14; 95% CI -1.25 to 0.97; *p* = 0.75; I² = 90.64%). (Fig. 14) This finding remained non-significant in all leave-one-out sensitivity analyses. A subgroup analysis at 3-month and 1-year follow-ups revealed no differences. (Fig. 15) Another subgroup analysis by robotic systems further supported the null effect, revealing no significant difference with NAVIO (MD -0.40; *p* = 0.78) or YUANHUA (MD 0.04; *p* = 0.95). (Fig. 16)

### Secondary Outcomes

The pooled analysis of KSS satisfaction score (4 studies, *n* = 445; RA-TKA = 221, C-TKA = 224) revealed no statistically significant variation between the two surgical approaches, with a pooled mean difference (MD) of 0.74 (95% CI − 4.42 to 5.90; *p* = 0.68; I² = 81.78%). (Supplementary Fig. S1) The pooled MD remained non-significant in all leave-one-out sensitivity analyses, confirming the null finding.

The meta-analysis of EuroQol 5-Dimension (EQ-5D) (4 studies, *n* = 290; RA-TKA = 150, C-TKA = 140) did not reveal a significant difference between the two surgical approaches, with a pooled MD of 0.04 (95% CI − 0.03 to 0.11; *p* = 0.16; I² = 43.29%). (Supplementary Fig. S2) Leave-one-out sensitivity analysis identified Vermue et al., [32] as a major source of heterogeneity; its exclusion reduced I² to 0.00% and rendered the result statistically significant (MD 0.05; 95% CI 0.01 to 0.09; *p* = 0.028). For the 36-Item Short Form Health Survey (SF-36) (3 studies, *n* = 333; RA-TKA = 165, C-TKA = 168), the pooled analysis remained non-significant in the primary analysis and in all leave-one-out scenarios, with a pooled MD of 0.73 (95% CI − 13.78 to 15.23; *p* = 0.85; I² = 13.31%). (**Supplementary Fig. S3**)

A pooled analysis of postoperative C-reactive protein (CRP) levels (3 studies, *n* = 165; RA-TKA = 82, C-TKA = 83) was performed separately for each follow-up time point due to differing reporting periods. The pooled analysis revealed no statistically significant differences at any follow-up, supporting the null finding. (**Supplementary Fig. S4**)

The pooled analysis of operative time (14 studies, *n* = 2673; RA-TKA = 1376, C-TKA = 1297) demonstrated significantly longer duration in the RA-TKA group, with a pooled mean difference (MD) of 22.34 min (95% CI 13.66 to 31.02; *p* < 0.01; I² = 98.53%). (**Supplementary Fig. S5**) The pooled MD remained statistically significant in all leave-one-out sensitivity analyses; however, heterogeneity exceeded 98% across all scenarios, highlighting caution in interpreting this finding. Moreover, subgroup analyses revealed that all robotic systems were associated with longer operative times compared to C-TKA. However, none of the analyses were statistically significant: ROBODOC (MD 17.19 min; *p* = 0.21), EPMEDBOT (MD 32.96 min; *p* = 0.14), NAVIO (MD 16.27 min; *p* = 0.13), and YUANHUA (MD 25.75 min; *p* = 0.30). **(Supplementary Fig. S6**)

The meta-analysis of length of hospital stay (LOS) (6 studies, *n* = 622; RA-TKA = 352, C-TKA = 270) revealed no statistically significant variation between the two surgical approaches, with a pooled mean difference (MD) of -0.12 days (95% CI − 0.81 to 0.58; *p* = 0.68; I² = 83.99%). (**Supplementary Fig. S7**) The pooled MD remained non-significant in all leave-one-out sensitivity analyses, confirming the robustness of the null finding.

The pooled analysis of intraoperative blood loss (7 studies, *n* = 1875; RA-TKA = 936, C-TKA = 939) revealed no significant difference between the two surgical approaches, with a pooled mean difference (MD) of 4.89 mL (95% CI − 11.46 to 21.25; *p* = 0.49; I² = 57.81%). (**Supplementary Fig. S8**) The pooled MD remained non-significant in all leave-one-out sensitivity analyses, confirming the robustness of the null finding.

The pooled analysis of postoperative drainage volume (3 studies, *n* = 1481; RA-TKA = 741, C-TKA = 740) revealed no significant difference between the two surgical approaches, with a pooled mean difference of − 88.94 mL (95% CI − 542.85 to 364.98; *p* = 0.49; I² = 89.97%). (**Supplementary Fig. S9**) Furthermore, the pooled analysis remained statistically insignificant in all leave-one-out scenarios, supporting the null finding.

Total blood loss was reported in 2 studies involving 172 patients: 90 in the RA-TKA group and 82 in the C-TKA group. A random-effects meta-analysis demonstrated a pooled MD of -73.65 mL (95% CI: -100.3 to -47.01; *p* = 0.02; I² = 0.00%), which is statistically significant. (**Supplementary Fig. S10**) However, this finding should be interpreted with caution due to the limited number of contributing studies. Postoperative hemoglobin changes were reported in 2 studies involving 248 patients: 164 in the RA-TKA group and 84 in the C-TKA group. The pooled analysis demonstrated a pooled MD of -0.77 (95% CI: -3.94 to 2.41; *p* = 0.20; I² = 31.03%), which is not statistically significant. (**Supplementary Fig. S11**)

### Trial sequential analysis

To further assess whether the current evidence for operative time is conclusive despite the high heterogeneity, we performed a Trial Sequential Analysis. Based on the variance derived from low‑risk studies and heterogeneity correction, the required information size (RIS) was estimated at 3250 patients. The cumulative Z‑curve crossed both the conventional boundary and the trial sequential monitoring boundary, suggesting that the observed difference is unlikely to be attributable to random error. However, the cumulative Z‑curve did not reach the RIS, indicating that the required information size has not yet been fully attained. Therefore, while crossing the monitoring boundaries provides statistical support for the finding that RA‑TKA is associated with longer operative time than C‑TKA, the evidence should be interpreted with caution. Future research, particularly involving surgeons with greater experience and after they have overcome the learning curve associated with robotic systems, may yield different estimates and potentially alter the current observation. (**Supplementary Fig. S12)**

### Meta-regression analysis

Meta‑regression for change in WOMAC function score included 9 studies for mean age, proportion of females, and follow‑up duration, and 8 studies for mean BMI. None of the examined covariates significantly influenced the effect estimates. The coefficients were as follows: mean age (coefficient: -0.31, 95% CI: -3.31 to 2.68, *p* = 0.81), mean BMI (coefficient: 0.54, 95% CI: -4.61 to 5.70, *p* = 0.80), proportion of females (coefficient: -0.017, 95% CI: -0.30 to 0.27, *p* = 0.89), and follow‑up duration (coefficient: 0.13, 95% CI: -0.13 to 0.40, *p* = 0.28). The residual I² values ranged from 62.08% to 80.57%, and the R² values indicated that the covariates explained 0% to 27.84% of the between‑study variance. (**Supplementary Table S7**)

For change in KSS function score, meta‑regression included 12 studies for mean age and follow‑up duration, and 11 studies for mean BMI and proportion of females. None of the covariates reached statistical significance. The coefficients were: mean age (coefficient: -0.21, 95% CI: -1.92 to 1.49, *p* = 0.78), mean BMI (coefficient: 0.33, 95% CI: -2.14 to 2.81, *p* = 0.76), proportion of females (coefficient: -0.18, 95% CI: -0.40 to 0.04, *p* = 0.09), and follow‑up duration (coefficient: 0.10, 95% CI: -0.46 to 0.24, *p* = 0.52). The residual I² values ranged from 74.35% to 82.56%, with R² values indicating that the covariates explained 0% to 27.84% of the between‑study variance. (**Supplementary Table S8**)

Meta‑regression for operative time included 14 studies for mean age, 13 studies for proportion of females, and 12 studies for mean BMI. Among all covariates examined, only mean BMI demonstrated a statistically significant association with operative time (coefficient: -7.84, 95% CI: -15.16 to -0.53, *p* = 0.038), suggesting a potential moderating effect. Mean age (coefficient: 0.59, 95% CI: -2.60 to 3.79, *p* = 0.69) and proportion of females (coefficient: -0.12, 95% CI: -0.60 to 0.35, *p* = 0.58) were not significant predictors. The residual I² values remained high across models (97.53% to 98.19%), with R² values ranging from 0% to 30.77%. (**Supplementary Table S9**)

According to established methodological guidance, meta‑regressions with a limited number of studies are more prone to false findings and may produce unreliable estimates. Therefore, these results should be interpreted with caution and considered hypothesis‑generating rather than conclusive evidence of sources of heterogeneity. They point to potential areas for future research but do not confirm definitive explanations. Overall, with the exception of a potential moderating effect of mean BMI on operative time, none of the examined covariates consistently explained the observed heterogeneity across the three outcomes, suggesting that other unmeasured factors may contribute to the between‑study variability.

### Publication bias assessment and influence diagnostics

Funnel plots and Egger’s regression test were used to assess publication bias for outcomes with 10 or more included studies. These outcomes included changes in KSS function score and operative time. For the change in KSS function score, the funnel plot demonstrated relative symmetry, and Egger’s test showed no significance (*SE = 1.28;p = 0.0872*), suggesting no evidence of publication bias. For operative time, the funnel plot showed some asymmetry, but Egger’s test was not significant (SE = 2.70; *p* = 0.36), indicating no statistical evidence of publication bias despite the visual asymmetry. Influence diagnostics were performed using Baujat plots for outcomes with sufficient studies, including change in WOMAC function score, change in KSS function score, and operative time. These analyses identified Song et al., [29], and Clement et al., [36], as major contributors to heterogeneity in changes in WOMAC function score. Regarding change in KSS function, Bollars et al., [43], and Lychagin et al., [25], were identified as influential. For operative time, Baujat plot analysis revealed that multiple studies contributed to the observed heterogeneity, with no single study exerting excessive influence. All funnel plots, Egger’s test results, and Baujat plots are presented in the Supplementary File.

## Discussion

This comprehensive review, drawing data from 24 RCTs involving approximately 3,425 patients, represents one of the most extensive and contemporary evaluations of RA-TKA versus C-TKA, with a primary focus on functional outcomes and pain relief. By incorporating the latest high‑quality RCTs published up to 2025, our study updates and expands upon previous syntheses, many of which were limited by smaller sample sizes or earlier generations of robotic technology. A key strength of our updated synthesis is the use of meta‑regression to identify sources of heterogeneity and the application of the GRADE framework to evaluate the certainty of the evidence, thereby addressing important limitations of previous research. Furthermore, we performed subgroup analyses to evaluate outcomes by specific robotic system, a crucial step toward a more nuanced understanding of how different platforms perform. Importantly, our study further distinguishes itself from prior meta‑analyses by including only randomized controlled trials and focusing exclusively on change‑from‑baseline measures, which more accurately reflect the magnitude of patient improvement and account for potential differences in baseline characteristics across studies, thereby enhancing the clinical interpretability of recovery trajectories. However, before interpreting the pooled findings, it is essential to acknowledge the substantial clinical heterogeneity across the included studies. The trials varied considerably with respect to robotic platforms (e.g., MAKO, ROBODOC, NAVIO, EPMEDBOT, YUANHUA), surgical workflow, alignment strategies (mechanical vs. kinematic), implant characteristics (posterior‑stabilized vs. cruciate‑retaining), surgeon experience, learning curves, and perioperative protocols. This heterogeneity, while some of it was addressed through subgroup analyses and meta‑regression, inherently limits the clinical interpretability of aggregate pooled estimates and underscores the need for caution when drawing broad conclusions.

The pooled analysis of change in WOMAC function score demonstrated no significant difference between RA‑TKA and C‑TKA (MD 0.14; *p* = 0.96), with the null finding remaining consistent across all sensitivity analyses and follow‑up intervals. Similarly, the pooled analysis of change in KSS function score did not reveal a significant difference (MD 0.51; *p* = 0.78), and this null result was robust across all leave‑one‑out analyses, follow‑up points, and robotic platform subgroups. In addition, none of the mean differences for WOMAC and KSS functional scores met the MCID [45, 46]. Moreover, the meta‑analysis of change in OKS also demonstrated no statistically significant improvement (MD 2.07; *p* = 0.51), with the null finding persisting across all sensitivity analyses. Although a subgroup analysis by follow‑up intervals revealed a significant difference only at 6 months (MD 2.25; *p* = 0.01), this observed effect did not meet the MCID [47] and was not sustained at other time points, supporting the fragility of this temporary effect. These consistently null findings across all primary functional outcomes indicate that robotic assistance does not confer a clinically meaningful advantage in the magnitude of functional improvement from baseline compared to conventional TKA. The changes in WOMAC and OKS findings are supported by very low‑certainty evidence. Furthermore, the change in KSS at 3‑months is supported by low‑certainty evidence, while at 1‑year it is very low certainty. Therefore, all estimates of functional improvement should be interpreted with substantial caution. In prior literature, RA-TKA similarly showed no differences in WOMAC, KSS, or OKS scores when compared to C-TKA, which aligns with our null findings [48, 49].

The pooled analysis of range of motion (ROM) did not reveal significant differences between the two surgical techniques. The pooled mean difference for flexion was 0.65° (*p* = 0.80), with the null finding remaining consistent across all sensitivity analyses and follow‑up points. For extension ROM, the pooled analysis similarly showed no difference (MD 0.23°; *p* = 0.41). This equivalence likely arises because ROM is primarily determined by patient factors such as preoperative stiffness, rehabilitation adherence, and scar formation, rather than intraoperative precision alone. This finding aligns with several recent meta‑analyses, which similarly reported no overall superiority of ROM for RA‑TKA at mid‑term follow‑up [13, 48].

Pain outcomes, as measured by change in VAS pain score, demonstrated no differences between the two techniques (MD − 0.14; *p* = 0.75), with null findings across all sensitivity analyses, follow‑up intervals (*3 months and 1 year*), and robotic platform subgroups (NAVIO and YUANHUA). This consistent equivalence suggests that pain pathways after TKA are more influenced by multimodal analgesia, resolution of inflammation, and individual pain thresholds than by alignment accuracy. This is supported by multiple meta‑analyses, which suggest equivalent short‑ to mid‑term pain relief [49, 50, 51]. The evidence for change in VAS pain at 1‑year is rated as very low certainty, downgraded due to serious risk of bias, very serious inconsistency (I² = 90.64%), and serious imprecision, limiting confidence in this estimate.

Secondary outcomes demonstrated largely null findings. The pooled analysis of KSS satisfaction score revealed no significant difference (MD 0.74; *p* = 0.68), with the null finding remaining robust across all sensitivity analyses. This is supported by very low‑certainty evidence. The meta‑analysis of EQ‑5D also did not reveal a significant difference (MD 0.04; *p* = 0.16). The SF‑36 analysis remained non‑significant (MD 0.73; *p* = 0.85). CRP levels showed no significant differences at any follow‑up. The levels of most blood loss parameters (intraoperative loss, drainage volume, hemoglobin drop) were comparable, although total blood loss was modestly lower with RA‑TKA in the limited available data (− 73.65 mL; *p* = 0.02; I² = 0.00%, 2 studies, *n* = 172). However, intraoperative blood loss showed no significant difference (MD 4.89 mL; *p* = 0.49), supported by very low‑certainty evidence, and this estimate may be altered by further research. The modest total blood loss benefit may stem from avoiding femoral intramedullary rods in robotic systems, which reduces marrow bleeding and transfusion requirements in select cases, but this finding is limited by the small number of contributing studies.

The analysis of length of hospital stay revealed no significant difference (MD − 0.12 days; *p* = 0.68), and the null finding remained robust across all sensitivity analyses. This parity occurs because discharge criteria are now standardized around pain control, mobility milestones, and home support, which are not significantly altered by robotic assistance. This aligns with Sun et al., who also reported no significant difference (MD − 0.31 days; *p* = 0.07) [49]. However, this is contrary to Londhe et al. [52], who reported a consistent and statistically significant decrease in hospital stay associated with RA‑TKA, and Fontalis et al. [53], who confirmed shorter hospital stay with RA‑TKA. Our updated analysis does not support these previous observations, and the discrepancy likely reflects differences in study populations, healthcare settings, or the inclusion of non‑randomized data in prior reports.

The most notable and consistent observation in RA‑TKA remains the prolonged operative time, which is increased by a mean of 22.34 min (*p* < 0.01). This finding is both statistically and clinically significant and, importantly, favors conventional surgery. The pooled MD remained statistically significant in all leave‑one‑out sensitivity analyses; however, heterogeneity exceeded 98% across all scenarios, highlighting caution in interpreting the precise magnitude. In addition, subgroup analyses revealed that all robotic systems were associated with longer operative times compared to C‑TKA, though none of the subgroup analyses reached statistical significance: ROBODOC (MD 17.19 min; *p* = 0.21), EPMEDBOT (MD 32.96 min; *p* = 0.14), NAVIO (MD 16.27 min; *p* = 0.13), and YUANHUA (MD 25.75 min; *p* = 0.30), highliting the need for coution interpretetion of this finding. To further assess whether the current evidence for operative time is conclusive despite the high heterogeneity, we performed a Trial Sequential Analysis, and the required information size (RIS) was estimated at 3,250 patients. The cumulative Z‑curve crossed both boundaries, suggesting that the observed difference is unlikely to be attributable to random error. However, it did not reach the RIS, indicating that the required information size has not yet been fully attained. Therefore, while crossing the monitoring boundaries may provide statistical support for the finding that RA‑TKA may provide longer operative time than C‑TKA, the evidence should be interpreted with caution. Furthermore, the evidence for operative time is rated as very low certainty. A critical factor that may contribute to the observed longer operative time is the learning curve associated with adopting this technology. The included studies varied considerably in terms of surgeon experience, with some trials enrolling patients during the early adoption phase. Kayani et al. revealed that the learning curves for operative times and surgical team confidence were approximately 7 cases [54], while Sodhi et al. found that operative times were prolonged for the initial 20 robotic TKA cases; beyond this threshold, operative times became comparable to manual TKA [55]. In this meta‑analysis, we were unable to account for surgeon experience due to inconsistent reporting. Consequently, the pooled estimate of operative time may overestimate the true procedural duration for experienced robotic surgeons, as it includes cases performed during the early learning curve. Future research, particularly involving surgeons with greater experience and after they have overcome the learning curve, may yield different estimates and potentially alter the current observation. Therefore, the operative time penalty should be interpreted with consideration of surgeon proficiency, and future studies should report surgeons’ experience levels and evaluate outcomes stratified by learning curve phase.

Economically, Hua et al. indicated that RA‑TKA may be economically viable in high‑volume centers, with an ICER of $41,331 per QALY gained [56]. Similarly, Goudazi et al. reported that cost‑effectiveness depends on institutional case volume and regional healthcare cost structures [57]. However, the substantial capital investment, ongoing consumable costs, and longer operative time remain important economic considerations. These factors, combined with the lack of clinically meaningful functional superiority, suggest that the economic case for widespread adoption of RA‑TKA remains uncertain.

### Strengths and limitations

This meta‑analysis has several vital strengths that enhance its reliability and clinical relevance. First, the study was carried out following the PRISMA guidelines. It involved a comprehensive, pre‑registered search across multiple major databases (PubMed, Web of Science, Scopus, the Cochrane Library, and Google Scholar), yielding one of the largest pooled samples (*3*,*425 patients*) from 24 randomized controlled trials published through 2025. By restricting inclusion to RCTs only and performing a rigorous risk‑of‑bias assessment using the Cochrane RoB‑2, selection bias and confounding were minimized, providing the highest level of evidence for comparative effectiveness. The inclusion of recent trials evaluating diverse robotic systems from multiple continents improves generalizability across different healthcare settings and surgical workflows. Advanced statistical techniques, including random‑effects modeling with the REML estimator, Hartung‑Knapp‑Sidik‑Jonkman variance adjustment, leave‑one‑out sensitivity analyses for all outcomes with more than two studies, Baujat and influence diagnostics, and prespecified subgroup analyses by follow‑up duration, were systematically applied to explore and mitigate heterogeneity while ensuring the robustness of the pooled estimates. Crucially, we advanced our analysis by conducting a comprehensive meta‑regression to identify sources of heterogeneity and by using the GRADE framework to evaluate the certainty of the evidence, thereby offering a more nuanced interpretation of our findings. A further strength of this updated analysis is the exclusive focus on change‑from‑baseline measures, which more accurately capture the magnitude of patient improvement and account for variability in baseline characteristics across studies, providing a more clinically meaningful assessment of recovery trajectories. Additionally, we performed subgroup analyses by robotic system for key outcomes, which allowed us to move beyond the limitations of a “one‑size‑fits‑all” approach and identify potential platform‑specific effects. This directly addresses a major limitation of prior meta‑analyses and provides a more clinically relevant synthesis. Furthermore, we performed Trial Sequential Analysis (TSA) for operative time, an outcome characterized by very high heterogeneity, to assess whether the observed difference was robust against random error. By omitting high‑risk‑of‑bias studies from the variance estimation, we adopted a conservative approach to evaluate the conclusiveness of the evidence.

However, it is important to note several limitations. Significant statistical heterogeneity was observed in many functional and perioperative outcomes, primarily attributable to differences in robotic systems, alignment targets, implant designs, and varying levels of surgeon experience with robotics. While meta‑regression helped explore heterogeneity, significant heterogeneity remained unexplained for key outcomes, indicating the possible influence of unmeasured confounders. Importantly, as noted above, most meta‑regression findings are based on study‑level data and a small number of contributing studies; therefore, these results should be regarded as exploratory and primarily intended to generate hypotheses rather than establish firm conclusions. The substantial clinical heterogeneity across the included trials also means that the aggregated pooled findings should be considered carefully, as they may not be generalizable to all clinical settings or patient populations. Additionally, our study included several trials that were rated as high risk of bias in the quality assessment, which warrants caution when interpreting the results. This is reflected in the GRADE assessment, where the certainty varied between low and very low. Follow‑up in most included trials remains short to mid‑term (≤ 2 years), limiting firm conclusions regarding long‑term implant performance and survivorship, revision rates, and the potential clinical translation of any radiographic advantages. A potential unit‑of‑analysis limitation should also be acknowledged. One included RCT [28] reported outcomes at the knee level in patients undergoing simultaneous bilateral TKA. Although sensitivity analyses demonstrated that omitting this study did not materially affect the pooled estimates, the assumption of independence between knees may not hold, potentially leading to an underestimation of variance. Therefore, these observations should be considered within the context of the study limitations, and future studies are encouraged to report patient‑level data or provide sufficient information to allow appropriate statistical adjustment. Furthermore, inconsistent reporting on whether procedures were unilateral or bilateral TKA prevented subgroup analyses on this factor, which is important for future research to better understand potential differences in outcomes. In addition, the included studies did not consistently report surgeon experience or the phase of the learning curve for robotic‑assisted procedures. Given that operative time and potentially other outcomes may be influenced by surgeon proficiency with robotic systems, the absence of this information limits our ability to distinguish between the intrinsic effects of robotic technology and those attributable to the learning curve. This limitation should be considered, particularly for operative time and outcomes that may be sensitive to surgical efficiency. Finally, industry funding and blinding concerns inherent to this literature should be acknowledged, as they may introduce bias in favor of robotic systems. These limitations highlight the need for longer‑term, appropriately powered RCTs with standardized protocols to definitively clarify the role of RA‑TKA in contemporary practice.

### Clinical guidance and future research

The interpretation of our pooled findings should be considered in light of the substantial clinical heterogeneity across the included studies, including differences in robotic platforms, surgical workflows, alignment strategies, implant designs, surgeon learning curves, and follow‑up duration. Given this heterogeneity, the aggregate pooled estimates presented in this meta‑analysis should not be interpreted as universally applicable to all RA‑TKA systems or clinical scenarios. Instead, these findings provide a broad overview of the current evidence base, with platform‑specific subgroup analyses offering more targeted insights where data permit. Importantly, our findings do not support the routine adoption of RA‑TKA over C‑TKA for primary TKA, as the evidence demonstrates equivalent functional outcomes, pain relief, and most perioperative parameters, with a consistent operative time penalty favoring conventional surgery. The meta‑regression analyses conducted in this study are based on study‑level data and a limited number of included trials for certain covariates. As such, these analyses should be considered hypothesis‑generating rather than confirmatory, and their results should be interpreted with extreme caution.

Future research should address the current evidence gaps to clarify the actual value of RA‑TKA. First, adequately powered, multicenter RCTs with long‑term follow‑up of at least 10–15 years are essential to evaluate whether the radiographic precision observed with RA‑TKA results in durable reductions in revision risk, improved implant survivorship, and sustained patient‑reported benefits compared with C‑TKA. Such trials should also be designed to prospectively gather data on potential effect modifiers, such as BMI, to validate the hypothesis‑generating findings from our meta‑regression. Second, the platform‑specific differences observed for some outcomes warrant direct comparative trials of different robotic systems to determine whether observed differences are due to specific technology, associated surgical workflow, or other factors. Third, comprehensive health‑economic evaluations are urgently needed. These should incorporate capital and maintenance costs of robotic systems, consumables, prolonged operative time, learning‑curve effects, complication profiles, and downstream healthcare utilization to determine whether RA‑TKA is cost‑effective across different healthcare settings and at varying surgical volumes. Fourth, larger RCTs with standardized protocols for perioperative care, implant selection, alignment targets, and complication reporting are required to reduce heterogeneity and provide more definitive comparisons of short‑ and long‑term functional recovery, pain relief, and patient satisfaction. Future studies should also ensure consistent reporting of whether procedures are unilateral or bilateral TKA to enable meaningful subgroup analyses, as this factor could influence outcomes. Additionally, studies should report surgeons’ experience levels and stratify outcomes by learning curve phase to better isolate the true effect of robotic technology from that of surgeon proficiency. Until robust evidence from these priority areas is available, RA‑TKA should be considered a precision‑enhancing adjunct that may be useful in selected complex cases, but it should not be adopted as the routine standard of care for primary TKA.

## Conclusion

This updated meta‑analysis demonstrates that RA‑TKA does not confer clinically meaningful advantages over C‑TKA in functional improvement, pain relief, patient satisfaction, ROM, or most perioperative outcomes. All primary functional outcomes (change in WOMAC, KSS, and OKS) did not differ significantly between groups, with null findings remaining consistent across all sensitivity analyses, follow‑up intervals, and robotic platform subgroups. The few statistically significant findings, such as the 6‑month OKS improvement and total blood loss reduction, were based on limited data, did not meet MCID thresholds, or were not robust across sensitivity analyses. The GRADE assessment rated the certainty as low to very low for all outcomes assessed, limiting confidence in the precision and stability of the estimated effects. The only consistent and clinically meaningful signal in this analysis is the approximately 22‑minute longer operative time associated with RA‑TKA, which favors conventional surgery. Trial Sequential Analysis confirmed that this finding is unlikely to be attributable to random error, though the very high heterogeneity and failure to reach the required information size warrant caution regarding the precise magnitude. This prolongation of operative time should be interpreted with consideration of the learning curve, as the pooled estimate may overestimate the true duration in the hands of experienced robotic surgeons. Collectively, the evidence suggests that robotic assistance is not associated with clinically meaningful functional outcomes, which remain largely driven by patient and rehabilitative factors. Based on current evidence, RA‑TKA should be considered as a technological adjunct rather than a superior standard of care for TKA. Future adequately powered, multicenter RCTs with long follow‑up and standardized protocols are needed to definitively clarify the role of RA‑TKA in contemporary practice.

## Supplementary Information

Below is the link to the electronic supplementary material.

Supplementary Material 1

## Data Availability

All study-related data, including that which was produced and subjected to analysis, are accessible in the final published paper and its associated supplementary content.

## Abbreviations

BMI: Body Mass Index
CI: Confidence Interval
CRP: C-reactive protein
C-TKA: Conventional Total Knee Arthroplasty
EQ-5D: EuroQol 5-Dimension
GRADE: Grading of Recommendations Assessment, Development, and Evaluation
HSS: Hospital for Special Surgery (score)
ICER: Incremental Cost-Effectiveness Ratio
KSS: Knee Society Score
LOS: Length of Stay
MD: Mean Difference
NR: Not reported
OKS: Oxford Knee Score
PRISMA: Preferred Reporting Items for Systematic Reviews and Meta-Analyses
RA-TKA: Robotic-Assisted Total Knee Arthroplasty
RCT: Randomized Controlled Trial
RoB-2: Cochrane Risk of Bias 2
ROM: Range of Motion
SD: Standard Deviation
SF-36: 36-Item Short Form Health Survey
VAS: Visual Analog Scale
WOMAC: Western Ontario and McMaster Universities Osteoarthritis Index
MCID: minimal clinically important differences
