# Supplementary material for: Comparative pain relief and functional outcomes following robotic-assisted versus conventional total knee arthroplasty: a GRADE-assessed meta-analysis with meta-regression of randomized controlled trials
Source: J Robot Surg. 2026 Sep 25;20(1):1021. doi: 10.1007/s11701-026-03934-0 (PMC13615089; doi:10.1007/s11701-026-03934-0)
Supplement: Supplementary file 1 [file 11701_2026_3934_MOESM1_ESM.docx]

**Supplementary File**

**Legends:**

A. **Tables**

**Supplementary Table S1**: The full search strings, including Boolean operators for each included database.

**Supplementary Table S2**: Assessment of potential patient overlap between Liow et al. 2014 and Liow et al. 2016.

**Supplementary Table S3:** Assessment of potential patient overlap between Song et al. 2011 and Song et al. 2013.

**Supplementary Table S4**: Assessment of potential patient overlap between Xu et al. 2022a and Xu et al. 2022b.

**Supplementary Table S5**: Assessment of potential patient overlap between Clement et al. 2023 and Clement et al. 2025.

**Supplementary Table S6**: Assessment of potential patient overlap between Geng et al. 2024 and Geng et al. 2025.

**Supplementary Table S7**: Meta-regression of selected covariates for the change in WOMAC functional score between RA-TKA and C-TKA at the last follow-up.

**Supplementary Table S8**: Meta-regression of selected covariates for the change in KSS functional score between RA-TKA and C-TKA at the last follow-up.

**Supplementary Table S9**: Meta-regression of selected covariates for operative time between RA-TKA and C-TKA.

B. **Figures**
1. **Forest Plots of Secondary Outcomes:**

**Supplementary Fig. S1.** Forest plot of postoperative KSS satisfaction score between RA-TKA and C-TKA at final follow-up.

**Supplementary Fig. S2**. Forest plot of postoperative EQ-5D score between RA-TKA and C-TKA at final follow-up.

**Supplementary Fig. S3.** Forest plot of postoperative SF-36 health survey between RA-TKA and C-TKA at final follow-up.

**Supplementary Fig. S4.** Forest plot of subgroup analysis of postoperative CRP levels between RA-TKA and C-TKA across distinct follow-up intervals.

**Supplementary Fig. S5**. Forest plot of operative time between RA-TKA and C-TKA.

**Supplementary Fig. S6.** Forest plot showing the subgroup analysis by robotic platform of operative time between RA-TKA and C-TKA.

**Supplementary Fig. S7.** Forest plot of length of stay (LOS) between RA-TKA and C-TKA.

**Supplementary Fig. S8.** Forest plot of intraoperative blood loss between RA-TKA and C-TKA.

**Supplementary Fig. S9.** Forest plot of postoperative drainage volume between RA-TKA and C-TKA.

**Supplementary Fig. S10.** Forest plot of total blood loss between RA-TKA and C-TKA.

**Supplementary Fig. S11.** Forest plot of hemoglobin change between RA-TKA and C-TKA.

2. **Trial Sequential Analysis**

**Supplementary Fig. S12**. Forest plot of Trial Sequential Analysis (TSA) of operative time between RA-TKA and C-TKA.

3. **Leave-one-out plots:**

**Supplementary Fig. S13.** Leave-one-out plot of postoperative WOMAC function score changes between RA-TKA and C-TKA at final follow-up.

**Supplementary Fig. S14.** Leave-one-out plot of postoperative KSS function score changes between RA-TKA and C-TKA at final follow-up.

**Supplementary Fig. S15.** Leave-one-out plot of postoperative OKS function score changes between RA-TKA and C-TKA at final follow-up.

**Supplementary Fig. S16.** Leave-one-out plot of postoperative flexion ROM between RA-TKA and C-TKA at final follow-up.

**Supplementary Fig. S17.** Leave-one-out plot of postoperative extension ROM between RA-TKA and C-TKA at final follow-up.

**Supplementary Fig. S18.** Leave-one-out plot of postoperative HSS score between RA-TKA and C-TKA at final follow-up.

**Supplementary Fig. S19.** Leave-one-out plot of postoperative VAS pain score changes between RA-TKA and C-TKA at final follow-up.

**Supplementary Fig. S20.** Leave-one-out plot of postoperative KSS satisfaction score between RA-TKA and C-TKA at final follow-up.

**Supplementary Fig. S21**. Leave-one-out plot of postoperative EQ-5D score between RA-TKA and C-TKA at final follow-up.

**Supplementary Fig. S22**. Leave-one-out plot of postoperative SF-36 score between RA-TKA and C-TKA at final follow-up.

**Supplementary Fig. S23.** Leave-one-out plot of operative time between RA-TKA and C-TKA.

**Supplementary Fig. S24.** Leave-one-out plot of length of stay (LOS) between RA-TKA and C-TKA.

**Supplementary Fig. S25.** Leave-one-out plot of intraoperative blood loss between RA-TKA and C-TKA.

**Supplementary Fig. S26.** Leave-one-out plot of postoperative drainage volume between RA-TKA and C-TKA.

4. **Influence Diagnostics, Funnel Plots, and Egger’s Regression**:

**Supplementary Fig. S27.** Baujat plot of change in WOMAC functional score between RA-TKA and C-TKA at the last follow-up.

**Supplementary Fig. S28**. Funnel plot of publication bias for postoperative KSS function score changes between RA-TKA and C-TKA at final follow-up.

**Supplementary Fig. S39**. Egger’s regression test of funnel plot asymmetry for change in KSS function score between RA-TKA and C-TKA.

**Supplementary Fig. S30.** Baujat plot of change in KSS functional score between RA-TKA and C-TKA at last follow-up.

**Supplementary Fig. S31**. Funnel plot of publication bias for operative time between RA-TKA and C-TKA.

**Supplementary Fig. S32**. Egger’s regression test of funnel plot asymmetry for operative time between RA-TKA and C-TKA.

**Supplementary Fig. S33.** Baujat plot of operative time between RA-TKA and C-TKA.

5. **Bubble Plots of Meta-regression:**

**Supplementary Fig. S34**. Bubble plot of mean age in the change in WOMAC functional score at the last follow-up.

**Supplementary Fig. S35.** Bubble plot of mean BMI in the change in WOMAC functional score at the last follow-up.

**Supplementary Fig. S36.** Bubble plot of the proportion of females (%) in the change in WOMAC functional score at the last follow-up.

**Supplementary Fig. S37.** Bubble plot of follow-up in the change in WOMAC functional score.

**Supplementary Fig. S38**. Bubble plot of mean age in the change in KSS functional score at the last follow-up.

**Supplementary Fig. S39.** Bubble plot of mean BMI in the change in KSS functional score at the last follow-up.

**Supplementary Fig. S40.** Bubble plot of the proportion of females (%) in the change in KSS functional score at the last follow-up.

**Supplementary Fig. S41**. Bubble plot of follow-up in the change in KSS functional score.

**Supplementary Fig. S42.** Bubble plot of mean age in the change in operative time.

**Supplementary Fig. S43**. Bubble plot of mean BMI in the change in operative time.

**Supplementary Fig. S44**. Bubble plot of the proportion of females (%) in operative time.

Supplementary Table S1: The full search strings, including Boolean operators for each included database.

| **Database** | **Search Strings** | **Records** |
| --- | --- | --- |
| PubMed | (“Robot”[tiab] OR “Robots”[MeSH] OR “Robotic”[tiab] OR “Robotic Assisted”[tiab] OR “Robotic-Assisted”[tiab] OR MAKO[tiab] OR NAVIO[tiab] OR ROSA[tiab] OR CORI[tiab] OR OMNIBotics[tiab]) AND (“Arthroplasty, Replacement, Knee”[MeSH] OR “Total Knee Arthroplast*”[tiab] OR TKA[tiab] OR “Total Knee Replacement”[tiab] OR TKR[tiab] OR “Knee Arthroplast*”[tiab] OR “Knee Replacement*”[tiab])) | 1580 |
| Scopus | TITLE-ABS-KEY(  (robot* OR "robotic assisted" OR "robotic-assisted"  OR MAKO OR NAVIO OR ROSA OR CORI OR OMNIBotics)  AND  ("total knee arthroplast*" OR TKA  OR "total knee replacement" OR TKR  OR "knee arthroplast*" OR "knee replacement*") | 1906 |
| Web of Science (WOS) | TS=(  (robot* OR "robotic assisted" OR "robotic-assisted"  OR MAKO OR NAVIO OR ROSA OR CORI OR OMNIBotics)  AND  ("total knee arthroplast*" OR TKA  OR "total knee replacement" OR TKR  OR "knee arthroplast*" OR "knee replacement*") | 1688 |
| Cochrane Library | (  (MeSH descriptor: [Robotics] explode all trees)  OR (robot* OR "robotic assisted" OR "robotic-assisted"  OR MAKO OR NAVIO OR ROSA OR CORI OR OMNIBotics)  )  AND  (  MeSH descriptor: [Arthroplasty, Replacement, Knee] explode all trees  OR ("total knee arthroplast*" OR TKA  OR "total knee replacement" OR TKR  OR "knee arthroplast*" OR "knee replacement*") | 223 |
| Google Scholar  *(pages 1-50)* | ("robotic assisted" OR "robotic-assisted" OR robot OR MAKO OR NAVIO OR ROSA OR CORI OR OMNIBotics)  AND  ("total knee arthroplasty" OR TKA OR "total knee replacement" OR TKR) | 1000 |

Supplementary Table S2: Assessment of potential patient overlap between Liow et al. 2014 and Liow et al. 2016.

| **Study ID** | **Sample Size** | | **Setting or Hospital** | **Patient recruitment time** | **Brief inclusion criteria** | **Key outcomes addressed** |
| --- | --- | --- | --- | --- | --- | --- |
|  | RA-TKA | C-TKA |  |  |  |  |
| Liow et al. 2014 | 31 | 29 | Singapore General Hospital | May  2012 to December 2012 | Primary knee osteoarthritis with genu varus deformity and a fixed flexion deformity of less than 15° | Oxford knee score, Length of Hospital Stay, and Operative Time. |
| Liow et al. 2016 | 31 | 29 | Singapore General Hospital | May  2012 to December 2012 | Diagnosis of primary knee  osteoarthritis with genu varus deformity and a fixed flexion deformity of less than 15° | Flexion and Extension Knee range of motion, Oxford knee score, Knee Society Function Score, and 36-Item Short Form Health Survey (SF-36) |

Supplementary Table S3: Assessment of potential patient overlap between Song et al. 2011 and Song et al. 2013.

| **Study ID** | **Sample Size** | | **Setting or Hospital** | **Patient recruitment time** | **Brief inclusion criteria** | **Key outcomes addressed** |
| --- | --- | --- | --- | --- | --- | --- |
|  | RA-TKA | C-TKA |  |  |  |  |
| Song et al. 2011 | 30 | 30 | Chonnam National University Hwasun  Hospital, Korea. | August 2004 to March  2006 | Patients with primary knee osteoarthritis and mechanical axis between 20° and 5° valgus, no previous hemiarthroplasty or total knee arthroplasty, no severe instability that could not be treated by cruciate-retaining TKA. | McMaster University (WOMAC) function scores. |
| Song et al. 2013 | 50 | 50 | Chonnam National  University Hwasun Hospital,  Korea. | July 2004 and September 2005 | Patients with primary knee osteoarthritis and mechanical axis between 20° and 5° valgus. | McMaster University (WOMAC) function scores, Hospital for Special Surgery (HSS) knee scores, operative time, and postoperative drainage. |

Supplementary Table S4: Assessment of potential patient overlap between Xu et al. 2022-1 and Xu et al. 2022-2.

| **Study ID** | **Sample Size** | | **Setting or Hospital** | **Patient recruitment time** | **Brief inclusion criteria** | **Key outcomes addressed** |
| --- | --- | --- | --- | --- | --- | --- |
|  | RA-TKA | C-TKA |  |  |  |  |
| Xu et al. 2022-1 | 17 | 16 | General  Hospital of PLA, Xiangya Hospital, Ruijin Hospital, West  China Hospital and Xinqiao Hospital of the Army Medical  University. | 2020 | Patients with end-stage osteoarthritis aged 18-80 years old with no intra-articular puncture or drug injection in the past 3 months and willing to give informed consent. | Hospital for Special Surgery (HSS) knee scores, VAS pain scores, C-reactive protein levels (CRP), postoperative hemoglobin change, postoperative drainage, and total blood loss. |
| Xu et al. 2022-2 | 37 | 35 | Chinese PLA General  Hospital | June to December 2020 | Patients with end-stage osteoarthritis aged 18-80 years. | Knee Society Score, C-reactive protein levels (CRP), operative time, Length of hospital stay, and intraoperative blood loss. |

Supplementary Table S5: Assessment of potential patient overlap between Clement et al. 2023 and Clement et al. 2025.

| **Study ID** | **Sample Size** | | **Setting or Hospital** | **Patient recruitment time** | **Brief inclusion criteria** | **Key outcomes addressed** |
| --- | --- | --- | --- | --- | --- | --- |
|  | RA-TKA | C-TKA |  |  |  |  |
| Clement et al. 2023 | 50 | 50 | Freeman  Hospital, Newcastle, UK | May 2019 to December 2021 | Patients with end-stage osteoarthritis aged 45-85 years, suitable candidates for cruciate-retaining TKA and under the care of two surgeons. | McMaster University (WOMAC) function scores, Oxford knee score. |
| Clement et al. 2025 | 22 | 18 | Freeman  Hospital, Newcastle, UK | May 2019 to December 2021 | Patients with end-stage osteoarthritis aged 45-85 years, suitable candidates for cruciate-retaining TKA and under the care of two surgeons. | McMaster University (WOMAC) function scores, Oxford knee score, EuroQol 5-Dimension (EQ-5D) scores. |

Supplementary Table S6: Assessment of potential patient overlap between Geng et al. 2024 and Geng et al. 2025.

| **Study ID** | **Sample Size** | | **Setting or Hospital** | **Patient recruitment time** | **Brief inclusion criteria** | **Key outcomes addressed** |
| --- | --- | --- | --- | --- | --- | --- |
|  | RA-TKA | C-TKA |  |  |  |  |
| Geng et al. 2024 | 65 | 65 | Peking University Third Hospital, The First Affiliated Hospital of Shandong First Medical University/ Shandong Provincial Qianfoshan Hospital, Jinan and  Chifeng Municipal Hospital, China. | November 2022 to May 2023 | Patients with knee osteoarthritis undergoing primary unilateral TKA aged ≥18 years and ≤80 years with written informed consent. | Knee Society Function Scores, 36-Item Short Form Health Survey (SF-36), Intraoperative Blood Loss. |
| Geng et al. 2025 | 73 | 66 | Peking University Third Hospital,  Tianjin Union Medical Center and Tianjin Fourth Central  Hospital, China. | February 2023 to May 2024 | Patients with knee osteoarthritis undergoing primary unilateral TKA aged ≥18 years and ≤80 years with written informed consent. | McMaster University (WOMAC) function scores, Knee society function scores, operative time and Total Blood Loss. |

Supplementary Table S7: Meta-regression of selected covariates for the change in WOMAC functional score between RA-TKA and C-TKA at the last follow-up.

| **Covariates** | **N** | **Coeff. (95%CI)** | **τ^2^** | **R^2^** | **I^2^** | **p-value** |
| --- | --- | --- | --- | --- | --- | --- |
| **Mean age (years)** | 9 | -0.31 (-3.31 to 2.68) | 46.22 | 0.00% | 78.28% | 0.81 |
| **Mean BMI** | 8 | 0.54 (-4.61 to 5.70) | 60.44 | 0.00% | 80.57% | 0.80 |
| **Proportion of females (%)** | 9 | -0.017 (-0.30 to 0.27) | 48.57 | 0.00% | 77.02% | 0.89 |
| **Follow-up duration (months)** | 9 | 0.13 (-0.13 to 0.40) | 27.55 | 27.84% | 62.08% | 0.28 |

Supplementary Table S8: Meta-regression of selected covariates for the change in KSS functional score between RA-TKA and C-TKA at the last follow-up.

| **Covariates** | **N** | **Coeff. (95%CI)** | **τ^2^** | **R^2^** | **I^2^** | **p-value** |
| --- | --- | --- | --- | --- | --- | --- |
| **Mean age (years)** | 12 | -0.21 (-1.92 to 1.49) | 34.48 | 0.00% | 82.56% | 0.78 |
| **Mean BMI** | 11 | 0.33 (-2.14 to 2.81) | 38.78 | 0.00% | 79.43% | 0.76 |
| **Proportion of females (%)** | 11 | -0.18 (-0.40 to 0.04) | 27.33 | 21.17% | 78.12% | 0.09 |
| **Follow-up duration (months)** | 12 | 0.10 (-0.46 to 0.24) | 33.07 | 27.84% | 74.35% | 0.52 |

Supplementary Table S9: Meta-regression of selected covariates for operative time between RA-TKA and C-TKA.

| **Covariates** | **N** | **Coeff. (95%CI)** | **τ^2^** | **R^2^** | **I^2^** | **p-value** |
| --- | --- | --- | --- | --- | --- | --- |
| **Mean age (years)** | 14 | 0.59 (-2.60 to 3.79) | 223.9 | 0.00% | 97.53% | 0.69 |
| **Mean BMI** | 12 | -7.84 (-15.16 to -0.53) | 155.96 | 30.77% | 98.19% | 0.038 |
| **Proportion of females (%)** | 13 | -0.12 (-0.60 to 0.35) | 181.4 | 0.00% | 97.62% | 0.58 |

1. **Forest Plots of Secondary Outcomes**:

Supplementary Fig. S1. Forest plot of postoperative KSS satisfaction score between RA-TKA and C-TKA at final follow-up.

Supplementary Fig. S2. Forest plot of postoperative EQ-5D score between RA-TKA and C-TKA at final follow-up.

Supplementary Fig. S3. Forest plot of postoperative SF-36 health survey between RA-TKA and C-TKA at final follow-up.

Supplementary Fig. S4. Forest plot of subgroup analysis of postoperative CRP levels between RA-TKA and C-TKA across distinct follow-up intervals.

Supplementary Fig. S5. Forest plot of operative time between RA-TKA and C-TKA.

Supplementary Fig. S6. Forest plot showing the subgroup analysis by robotic platform of operative time between RA-TKA and C-TKA.

Supplementary Fig. S7. Forest plot of length of stay (LOS) between RA-TKA and C-TKA.

Supplementary Fig. S8. Forest plot of intraoperative blood loss between RA-TKA and C-TKA.

Supplementary Fig. S9. Forest plot of postoperative drainage volume between RA-TKA and C-TKA.

Supplementary Fig. S10. Forest plot of total blood loss between RA-TKA and C-TKA.

Supplementary Fig. S11. Forest plot of hemoglobin change (g/dL) between RA-TKA and C-TKA.

2. **Trial Sequential Analysis (TSA)**

Supplementary Fig. S12. Forest plot of Trial Sequential Analysis (TSA) of operative time between RA-TKA and C-TKA.

3. **Leave-one-out plots**:

Supplementary Fig. S13. Leave-one-out plot of postoperative WOMAC function score changes between RA-TKA and C-TKA at final follow-up.

Supplementary Fig. S14. Leave-one-out plot of postoperative KSS function score changes between RA-TKA and C-TKA at final follow-up.

Supplementary Fig. S15. Leave-one-out plot of postoperative OKS function score changes between RA-TKA and C-TKA at final follow-up.

Supplementary Fig. S16. Leave-one-out plot of postoperative flexion ROM between RA-TKA and C-TKA at final follow-up.

Supplementary Fig. S17. Leave-one-out plot of postoperative extension ROM between RA-TKA and C-TKA at final follow-up.

Supplementary Fig. S18. Leave-one-out plot of postoperative HSS score between RA-TKA and C-TKA at final follow-up.

Supplementary Fig. S19. Leave-one-out plot of postoperative VAS pain score changes between RA-TKA and C-TKA at final follow-up.

Supplementary Fig. S20. Leave-one-out plot of postoperative KSS satisfaction score between RA-TKA and C-TKA at final follow-up.

Supplementary Fig. S21. Leave-one-out plot of postoperative EQ-5D score between RA-TKA and C-TKA at final follow-up.

Supplementary Fig. S22. Leave-one-out plot of postoperative SF-36 score between RA-TKA and C-TKA at final follow-up.

Supplementary Fig. S23. Leave-one-out plot of operative time between RA-TKA and C-TKA.

Supplementary Fig. S24. Leave-one-out plot of length of stay (LOS) between RA-TKA and C-TKA.

Supplementary Fig. S25. Leave-one-out plot of intraoperative blood loss between RA-TKA and C-TKA.

Supplementary Fig. S26. Leave-one-out plot of postoperative drainage volume between RA-TKA and C-TKA.

4. **Funnel Plots, Egger’s Regression, and Baujat Plots**:

Supplementary Fig. S27. Baujat plot of change in WOMAC functional score between RA-TKA and C-TKA at the last follow-up.

Supplementary Fig. S28. Funnel plot of publication bias for postoperative KSS function score changes between RA-TKA and C-TKA at final follow-up.

Supplementary Fig. S29. Egger’s regression test of funnel plot asymmetry for change in KSS function score between RA-TKA and C-TKA.

***Test result: t = 1.90, df = 10, p-value = 0.0872***

***Bias estimate: 2.4342 (SE = 1.2839)***

Supplementary Fig. S30. Baujat plot of change in KSS functional score between RA-TKA and C-TKA at the last follow-up.

Supplementary Fig. S31. Funnel plot of publication bias for operative time between RA-TKA and C-TKA.

Supplementary Fig. S32. Egger’s regression test of funnel plot asymmetry for operative time between RA-TKA and C-TKA.

***Test result: t = -0.94, df = 12, p-value = 0.3675***

***Bias estimate: -2.5344 (SE = 2.7066)***

Supplementary Fig. S33. Baujat plot of operative time between RA-TKA and C-TKA.

5. **Bubble Plots of Meta-regression**:

Supplementary Fig. S34. Bubble plot of mean age in the change in WOMAC functional score at the last follow-up.

Supplementary Fig. S35. Bubble plot of mean BMI in the change in WOMAC functional score at the last follow-up.

Supplementary Fig. S36. Bubble plot of the proportion of females (%) in the change in WOMAC functional score at the last follow-up.

Supplementary Fig. S37. Bubble plot of follow-up in the change in WOMAC functional score.

Supplementary Fig. S38. Bubble plot of mean age in the change in KSS functional score at the last follow-up.

Supplementary Fig. S39. Bubble plot of mean BMI in the change in KSS functional score at the last follow-up.

Supplementary Fig. S40. Bubble plot of the proportion of females (%) in the change in KSS functional score at the last follow-up.

Supplementary Fig. S41. Bubble plot of follow-up in the change in KSS functional score.

Supplementary Fig. S42. Bubble plot of mean age in the change in operative time.

Supplementary Fig. S43. Bubble plot of mean BMI in the change in operative time.

Supplementary Fig. S44. Bubble plot of the proportion of females (%) in operative time.
